# Hierarchical supercrystalline nanocomposites through the self-assembly of organically-modified ceramic nanoparticles

**DOI:** 10.1038/s41598-019-39934-4

**Published:** 2019-03-05

**Authors:** Berta Domènech, Michael Kampferbeck, Emanuel Larsson, Tobias Krekeler, Büsra Bor, Diletta Giuntini, Malte Blankenburg, Martin Ritter, Martin Müller, Tobias Vossmeyer, Horst Weller, Gerold A. Schneider

**Affiliations:** 1 Institute of Advanced Ceramics, Hamburg University of Technology, 21073 Hamburg, Germany; 2 Institute of Physical Chemistry, University of Hamburg, 20146 Hamburg, Germany; 3 Institute of Materials Research, Helmholtz-Zentrum Geesthacht, 21502 Geesthacht, Germany; 4 Electron Microscopy Unit, Hamburg University of Technology, 21073 Hamburg, Germany

## Abstract

Biomaterials often display outstanding combinations of mechanical properties thanks to their hierarchical structuring, which occurs through a dynamically and biologically controlled growth and self-assembly of their main constituents, typically mineral and protein. However, it is still challenging to obtain this ordered multiscale structural organization in synthetic 3D-nanocomposite materials. Herein, we report a new bottom-up approach for the synthesis of macroscale hierarchical nanocomposite materials in a single step. By controlling the content of organic phase during the self-assembly of monodisperse organically-modified nanoparticles (iron oxide with oleyl phosphate), either purely supercrystalline or hierarchically structured supercrystalline nanocomposite materials are obtained. Beyond a critical concentration of organic phase, a hierarchical material is consistently formed. In such a hierarchical material, individual organically-modified ceramic nanoparticles (Level 0) self-assemble into supercrystals in face-centered cubic superlattices (Level 1), which in turn form granules of up to hundreds of micrometers (Level 2). These micrometric granules are the constituents of the final mm-sized material. This approach demonstrates that the local concentration of organic phase and nano-building blocks during self-assembly controls the final material’s microstructure, and thus enables the fine-tuning of inorganic-organic nanocomposites’ mechanical behavior, paving the way towards the design of novel high-performance structural materials.

## Introduction

Natural biological materials, such as nacre or bone, are nanocomposites of proteins and minerals with simultaneously high toughness and strength^1–4^. Such outstanding properties are not simply due to the combination of optimal constituent materials, but mainly to their structural organization on multiple hierarchical levels. To do so, nature proceeds *via* the biologically controlled growth and self-assembly of the material’s main components, starting from the nanoscale. Recent advances in the development of bioinspired composites have aimed at controlling their structural features, especially at the microscale level, through top-down methods such as freeze casting and additive manufacturing^5^. Nevertheless, controlling the nanoscale *via* these top-down approaches has shown to be an intricate topic^6^. Bottom-up approaches such as self-assembly, open a new way for the development of hierarchical materials, allowing a better control starting from the nano-building blocks^7,8^. And although much has been done for the control of the nanoscale to obtain hierarchical nano- and microstructures using bottom-up approaches, its integration into large-scale materials is still an issue to overcome^7–14^. Thus, from a physico-chemical as well as an engineering viewpoint, the fabrication of 3D-hierarchically structured nanocomposite materials remains a big challenge.

Herein, we report the synthesis and characterization of a hierarchical nanocomposite material *via* the self-assembly of oleyl phosphate (OPh)-stabilized iron oxide (Fe_3_O_4_) nanoparticles (NPs). The synthetic approach is similar to what reported in a previous work published by our group using oleic acid as a ligand^10^. The main difference relies on the ligand shell: while oleic acid is liquid at room temperature, OPh remains in a condensed state, allowing the manufacturing of a 2-level hierarchical material. Even though the overall manufacturing process consists of 4 steps, the most challenging part, which is the hierarchical structuring, is achieved during the self-assembly by solvent evaporation. During this self-assembly process, the NPs’ surface modification by OPh, a relatively short aliphatic chain ligand, allows controlling the NPs’ arrangement by steric repulsion and interdigitation, counterbalanced by van der Waals attraction^15–17^. Moreover, the high monodispersity of the NPs (standard deviation of the size distribution below 5%) allows obtaining ordered and closely packed superstructures^10,18–21^. It will be shown that in such a material individual organically-modified nanoparticles (Level 0) self-assemble to form supercrystals (SCs) in a face-centered cubic (FCC) superlattice. These nucleated supercrystals grow to form micro-grains (Level 1), which finally constitute a mm-sized poly-supercrystalline material within an oleyl phosphate matrix phase (Level 2).

The results of this study show that with solvent evaporation it is possible to control the microstructure of the final material by tuning the local content of the organic phase. The size of the final SCs depends on the concentration of NPs in the suspension, which is constantly changing, because of the evaporation of the solvent and of the growth and sedimentation of the nucleated SCs. Finally, whether a Level 1 or Level 2 microstructure is formed depends on the concentration of OPh. If the amount of OPh is only enough to create a ligand monolayer shell on the NP surface, the final structure is a Level 1 poly-supercrystalline material. If there is an excess of OPh, a level 2 hierarchical material is obtained. Even if such an effect, to the best of our knowledge, has not yet been reported for self-assembled nanocomposites, this leads to the direct synthesis of a hierarchical supercrystalline material in one single step, with thus a high potential for the fine-tuning of the nanocomposites’ mechanical response.

## Results

### Preparation of oleyl phosphate-stabilized iron-oxide nanoparticles

**Figure 1:**
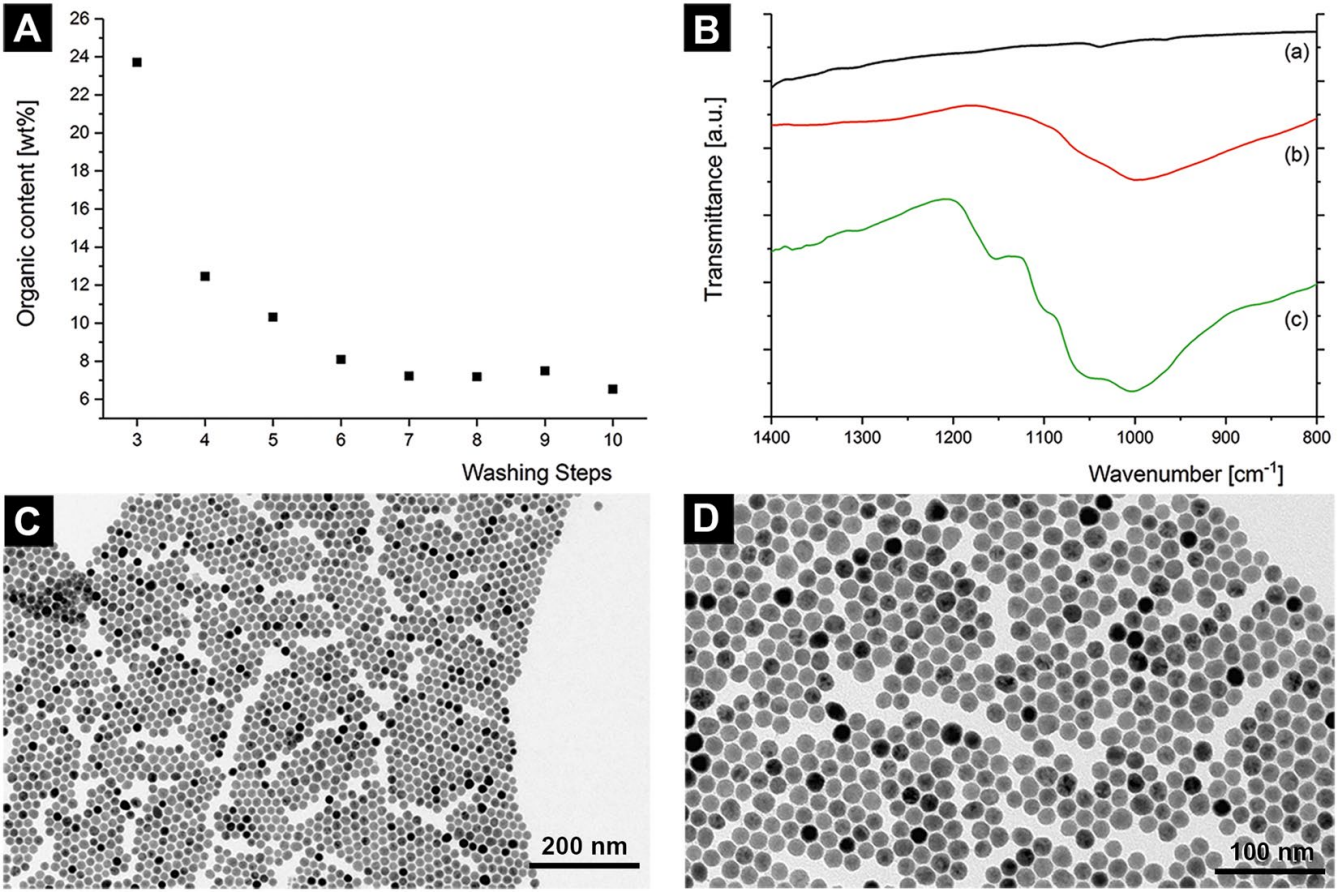
(**A**) Organic content after the washing steps, showing a reduction of the total organic content upon washing to a minimum of *approx*. 7 wt% determined by TGA. (**B**) Representative ATR-FTIR spectra within the most relevant wavenumber region (800–1400 cm^−1^) of iron oxide nanoparticles coated with (a) 9 wt% oleic acid, (b) 8 wt% oleyl phosphate (Batch 1), and (c) 21 wt% oleyl phosphate (Batch 2). From (a) to (c), strong bands are increasing in the region between 900–1250 cm^−1^, which are attributed to adsorbed phosphate at the surface of the nanoparticles. Full range overview spectra and spectra of the pure ligands, for comparison, can be found in the Fig. SI5. (**C**) TEM image of iron oxide nanoparticles stabilized with 8 wt% oleyl phosphate (Batch 1). (**D**) TEM image of iron oxide nanoparticles stabilized with 21 wt% oleyl phosphate (Batch 2).

Oleic acid-stabilized iron oxide nanoparticles (Fe_3_O_4_-OA NPs) were modified *via* a ligand exchange reaction, wherein oleic acid was replaced by oleyl phosphate (OPh) with a mixture of mono- and di-ester OPh (see Methods section). A series of washing steps after the ligand exchange allowed to efficiently remove the excess ligand. The amount of OPh remaining after each washing step was evaluated by Thermogravimetric Analysis (TGA) (Fig. 1A and Fig. SI2), and it was found that after the first seven washing steps the organic content was reduced to *approx*. 7 wt%. Moreover, additional washing steps after the seventh step did not further reduce the organic content in the sample, which indicates that this amount may correspond to a monolayer of stabilizing ligands bound to the surface of the particles.

In order to evaluate the effect of the organic content on the final materials obtained, two different batches were synthesised: Batch 1, with an organic content determined by TGA of 8 wt%, and Batch 2, with an organic content of 21 wt% (Fig. SI3). Both batches consisted of monodisperse nanoparticles with mean diameters of 18.4 ± 0.1 nm (Batch 1) and 18.6 ± 0.1 nm (Batch 2), as determined by high energy Small Angle X-ray Scattering (SAXS) (Fig. SI4). Note that the differences observed in the diameter of the nanoparticles from the starting suspension (Fig. SI1) *versus* Batch 1 and Batch 2 may be attributed to the different techniques used for the measurement^22,23^.

Attenuated Total Reflectance-Fourier-transform Infrared spectra (ATR-FTIR) of the samples after the ligand exchange (Fig. 1B and Fig. SI5) show four strong bands between 1250 and 900 cm^−1^ (located at 1152 cm^−1^, 1095 cm^−1^, 1050 cm^−1^, and 1000 cm^−1^) that are attributed to adsorbed phosphate on an iron oxide surface, while oleic acid does not show strong absorption in this region^24,25^.

The presence and the type (mono- or di-ester) of OPh in the final NPs’ suspensions used for this study (Batch 1 and 2) were also investigated by elemental analysis (SI, Chapter 2). For Batch 1 a grafting density of phosphate onto the iron oxide surface of 3.9 molecules/nm^2^ was found, being this density only somewhat higher than the previously reported grafting density of *approx*. 3.3 phosphate molecules/nm^2^ on magnetite surfaces and close to the theoretically determined anchoring area per phosphate molecule of 0.24 nm^2^ (4.2 molecules/nm^2^)^24^. Therefore, in Batch 1 the iron oxide NPs are surrounded by a covalently bonded monolayer of OPh molecules. Moreover, since the measured C/P-ratio was 18.3/1.0, it can be stated that, from the original mixture of mono- and di-ester OPh used for the ligand exchange reaction, the monoester is the only one remaining on the surface of the iron oxide. For Batch 2, a grafting density of 10.7 molecules/nm^2^ was obtained, corresponding to an approximately two-fold excess of chemically non-bound oleyl phosphate ligands. The overall organic content was determined to be 32.2 wt%. Because all oleic acid ligands were removed after the ligand exchange, the measured C/P-ratio of 23.2/1.0 indicates that the organic phase in Batch 2 consists of 71% monoester and 29% diester.

Comparing the results of TGA and elemental analysis, the discrepancy between the obtained organic contents is striking. Most likely this discrepancy can be attributed to the thermal decomposition processes of the ligands upon heating during the TGA measurements previously reported in literature^26,27^. Thus, taking into account the above-mentioned uncertainties related to TGA measurements, together with the similarity of the area of the anchoring group determined by elemental analysis to the theoretically reported value^24^, we conclude that the organic content of the starting suspensions (Batch 1 and Batch 2) is more reliably represented by the values from the elemental analysis. Therefore, in the following, the elemental analysis values − 12.4 wt% OPh for Batch 1 and 32.2 wt% OPh for Batch 2 - are considered.

### Self-assembled organically-modified ceramic nanoparticles materials

**Figure 2:**
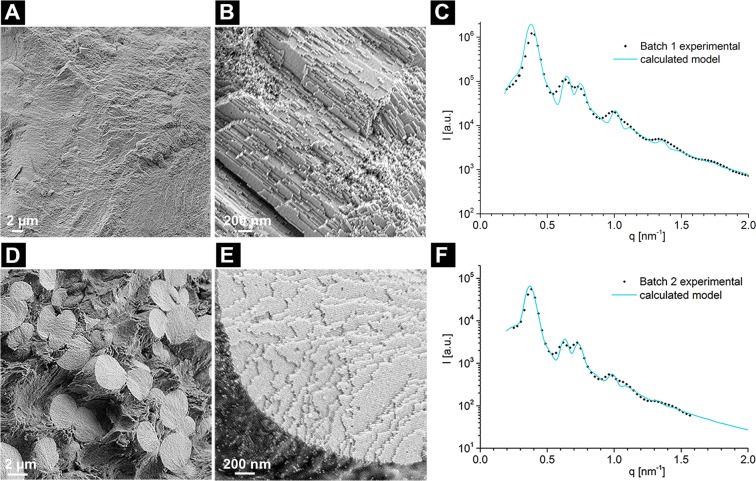
Scanning Electron Microscopy (SEM) images of the microstructure of the two different materials obtained with Batch 1 (**A**,**B**) and Batch 2 (**D**,**E**). The microstructure shown for Batch 2 is representative of the inner zone depicted in Fig. 3. Corresponding SAXS curves for Batch 1 (**C**) and for Batch 2 (**F**). The calculated model (continuous line) determined an FCC structure for both batches. More information is available in SI, Chapter 4.

To obtain the solid 3D- materials, suspensions of oleyl phosphate-coated iron oxide nanoparticles (Batch 1 or Batch 2) in toluene were self-assembled by solvent evaporation in a cylindrical vessel. The remaining dried sediments were then compacted *via* uniaxial pressing (see Methods section for more information about these steps). For both starting NPs suspensions, this procedure leads to 10-mm-sized pellets of ordered nanoparticles (Fig. 2). The pellets retained mechanical integrity, but were affected by cracks, most likely at the interfaces between different supercrystalline regions.

The SAXS investigation of samples obtained with both batches confirmed that both materials are formed by nanoparticles self-assembled into a face centred cubic (FCC) superlattice, with lattice parameters of 27.1 nm (Batch 1) and 28.2 nm (Batch 2), resulting in nearest neighbour distances (NND) of 19.2 (Batch 1) and 19.9 nm (Batch 2). These values correspond to interparticle distances (ID) of the Fe_3_O_4_-NPs of 0.8 ± 0.1 nm (Batch 1) and 1.3 ± 0.1 nm (Batch 2). If we assume that *ca*. 12 wt% (as determined for Batch 1) of the OPh is a homogeneous monolayer on the nanoparticle surface with a density of 0.95 g/cm^3^, a layer thickness of 1.9 nm is calculated (see SI, Chapter 3).

SEM evaluations of fracture surfaces of the samples allowed the determination of different microstructures for the materials obtained with the two starting batches. For Batch 1, with 12.4 wt% OPh, a supercrystalline (SC) bulk nanocomposite is obtained (Fig. 2A,B), similar to the ones obtained when using oleic acid as a stabilizing ligand on iron oxide NPs^10^. Instead, the sample obtained with the 32.2 wt% OPh content starting suspension (Batch 2) consists of microscopic spherical grains (Fig. 2D,E).

**Figure 3:**
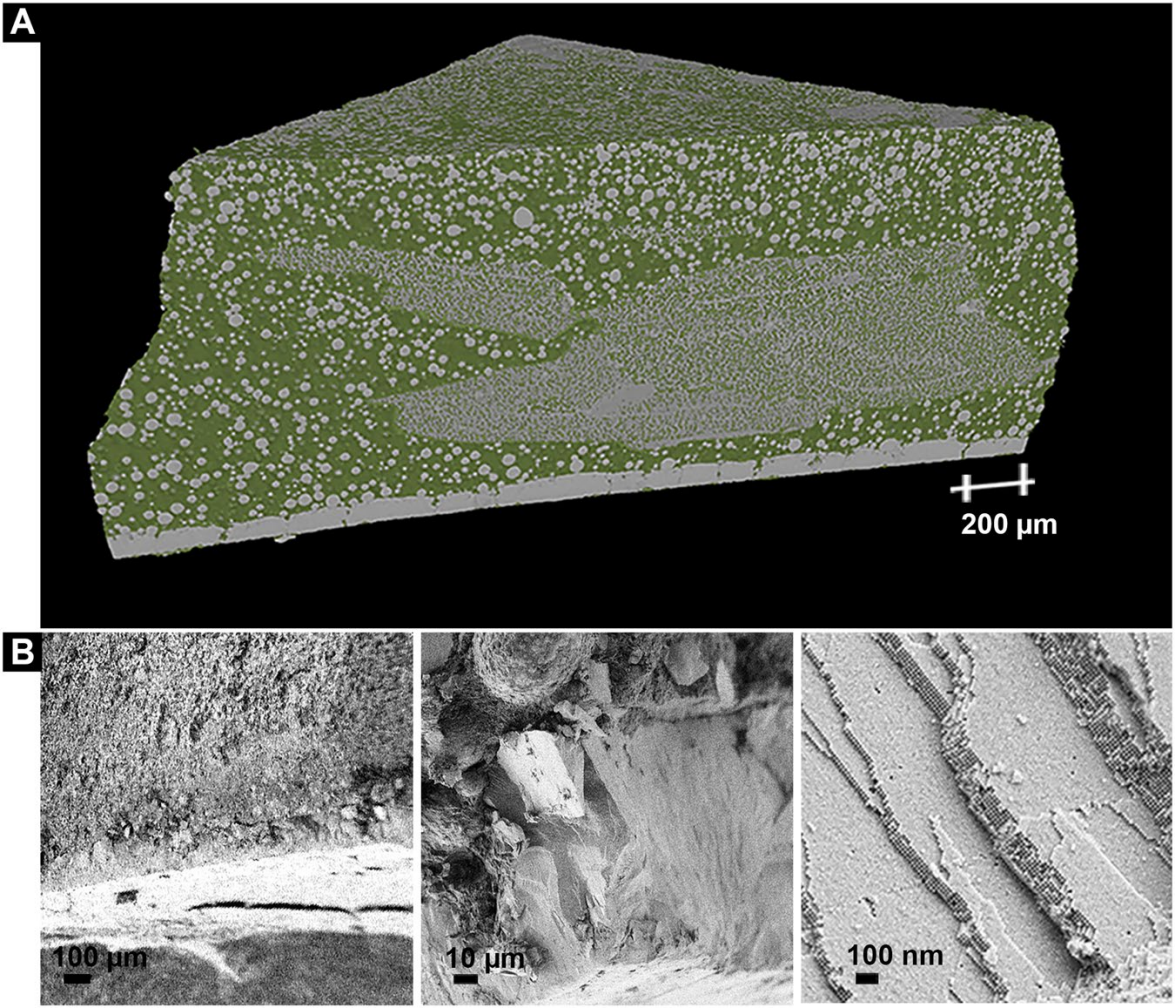
(**A**) Volume rendering of SRµCT data of a piece of Batch 2 material (32.3 wt% OPh) with the two phases being distinguished, high absorbing phase (in grey), and low absorbing phase (in green). (See Fig. SI8). (**B**) SEM images of the flat supercrystalline layer at the bottom of the sample. From left to right: zoomed-in views of the different areas from lower to higher magnification. SRµCT video of the whole sample is available as Supplementary video.

Further evaluation of the whole cross-section of a hierarchical sample obtained with Batch 2 by SEM and Synchrotron Micro-Tomography (SRµCT) (Fig. 3) shows that the material obtained is in fact composed by two different superstructures. At the bottom of the sample a flat layer 102 ± 6 µm thick (mean ± IC (95%) of 10 measurements) made of large flattened supercrystalline grains, is found. This layer is similar in microstructure to Batch 1. Above this flat supercrystalline layer a zone that extends 1014 ± 9 µm in the thickness direction (mean ± IC (95%) of 10 measurements), composed of supercrystalline micro- and nanosized grains surrounded by a matrix phase, appears. (All the above-mentioned measurements were performed with ImageJ (US National Institutes of Health, Bethesda, MD, USA)). Each of these grains is itself consisting of ordered nanoparticles in a FCC superlattice.

Moreover, SRµCT volume rendering of a piece of a Batch 2 sample (Fig. 3A and Supplementary video) shows that the sample is composed of two different materials’ phases, which could be separated based on their X-Ray linear attenuation coefficient (LAC) (in cm^−1^) as follows (see Fig. SI8): a high absorbing phase composed of supercrystalline grains, and a low absorbing phase mainly corresponding to the excess of organic material, probably mixed with very small supercrystalline grains with a size below the given resolution.

**Figure 4:**
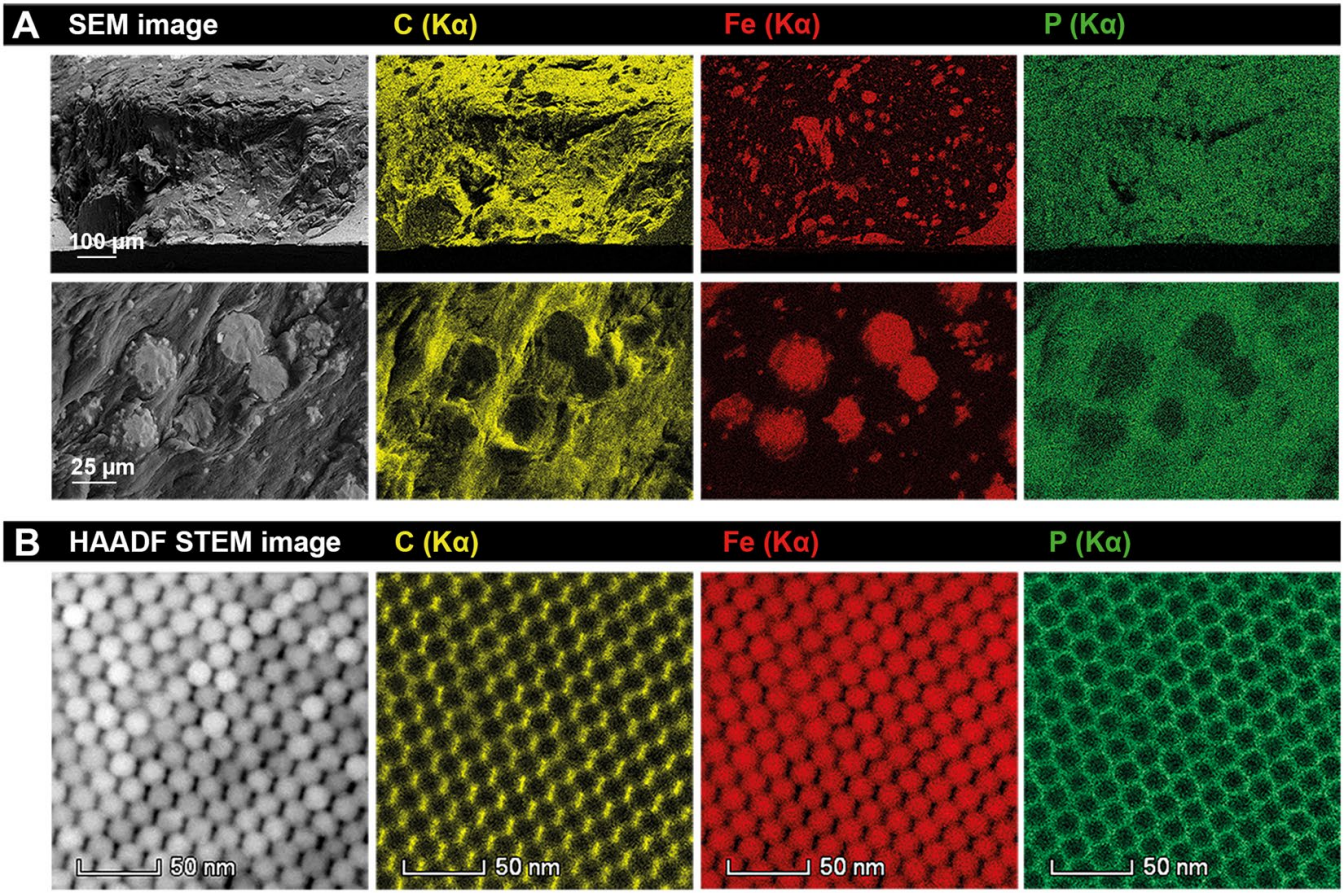
(**A**) SEM images of different areas of a sample prepared with Batch 2 and the corresponding spectrum images of carbon (yellow), iron (red), and phosphorous (green). (**B**) HAADF STEM image (left) and corresponding EDX of a supercrystalline grain (viewing direction [110]) from a FIB lamella of a sample obtained with Batch 2. In yellow, carbon; red, iron; and green, phosphorous.

SEM-Energy-Dispersive X-ray spectroscopy (EDX) was also used to determine the distribution of organic and inorganic material in the different parts of the nanocomposite material^28^. Elemental mapping on a fracture surface of the nanocomposite of Batch 2 (Fig. 4A) shows that iron is only present in the supercrystalline grains, while phosphorous and carbon appear over the entire analysed area, with less intensity in the supercrystalline grains. A Focused Ion Beam (FIB) lamella of a supercrystalline area of the nanocomposite obtained from Batch 2 was also evaluated by EDX. The results presented in Fig. 4B show that iron is mainly found in the nanoparticles core, while phosphorous and carbon appear on the nanoparticle shell. Note that, in Fig. 4B, the carbon distribution seems to be anisotropic around the NP’s core, but this effect is due to an artefact of the technique associated with the strong absorption of soft carbon X-Rays (280 eV) in iron oxide^29^.

**Figure 5:**
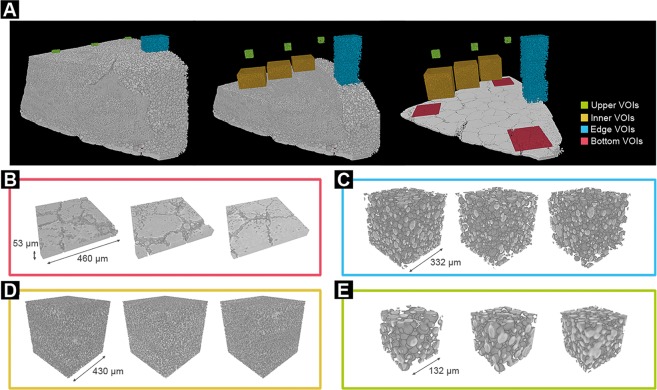
(**A**) Volume rendering of SRµCT data of a piece of a Batch 2 sample (with the edge of the sample being on the right side) and VOIs extracted from the different regions selected. 3D-renderings of VOIs extracted from (**B**) the flat supercrystalline bottom layer, (**C**) the outer edge of the sample at an increasing distance from the bottom of the sample (from left to right), (**D**) the outer edge of the sample at an increasing distance from the edge of the sample (from left to right), and (**E**) the upper part of the sample at an increasing distance from the edge of the sample (from left to right).

In order to better determine the structural characteristics of the sample, the SRµCT data was further evaluated by 3D-rendering of the strongly absorbing phase (associated with the supercrystalline material) and selecting representative Volume-of-Interests (VOIs), which were used for quantitative 3D analysis. Fig. 5 shows an overview of the sample and the VOIs extracted from the 4 different selected areas: bottom layer, edge (perimeter), inner part, and upper part of the sample. For the sake of simplicity, only 3 VOIs per selected region are shown. However, for the quantitative analysis and for the extraction of mean values and standard deviations, several VOIs per region were considered, all extracted based on a test of representative volume (SI, Chapter 6).

As shown in Fig. 5A,B, the bottom part of the sample is mainly composed by a flat layer of strongly absorbing material. Taking a closer look at the VOIs presented from this region (Fig. 5B), grain boundaries between the flat platelet-like grains appear, and few smaller spherical grains within these boundaries can be seen. Moreover, the quantitative analysis (Table 1) of the 3 VOIs extracted from this region (2 close to the edge of the sample and 1 in the inner part) shows that these selected volumes do not present any large differences in terms of composition, and they are mainly formed of flat grains with 89.6 vol% of high absorbing material (SC), and correspondingly 10.4 vol% of low absorbing material (organic-rich).

**Table 1:** Results of the quantitative analysis of the VOIs extracted from Volume rendering of SRµCT data of a piece of a Batch 2 sample.

	Bottom VOIs*	Edge VOIs			Upper VOIs**		
Distance to bottom (mm)	0	0.4	0.7	1.1	1	1	1
Distance to edge (mm)	—	0	0	0	0.1	1	1.8
Volume % of SC phase	89.6 ± 2.9	38 ± 0.5	30.2 ± 0.5	29.1 ± 0.3	33.7	48.3	58.3
Connectivity density***	—	19.3 ± 2.3 10^3 ^mm^−3^	2.4 ± 0.7 10^3^ mm^−3^	0.9 ± 0.4 10^3^ mm^−3^	1.1 10^7^ mm^−3^	6.2 10^7^ mm^−3^	20.2 10^7^ mm^−3^
Mean grain size (µm)	—	22.9 ± 8.8			18.5 ± 6.6	16.1 ± 5.0	13.4 ± 3.4 µm

*Since no significant differences between the 3 VOIs evaluated could be detected, only mean values are given, regardless from the distance to the edge.

**Since only 1 VOI per distance was evaluated, no standard deviations for the %volume and connectivity density are provided.

***Units for the connectivity density are indicated next to each value, being either 10^3^ mm^−3^ or 10^7^ mm^−3^.

Contrarily, the microstructure observed from the VOIs at the edge of the sample does present some differences depending on the position of the VOIs (in blue in Fig. 5A,C). In this region, when increasing the distance from the bottom of the sample, the % in volume of the SC phase decreases. Furthermore, the extracted image skeleton (Fig. SI11) from these VOIs show that grains are inter-connected (within the resolution of the SRµCT) and that the connectivity density decreases with the distance from the bottom layer. The Blob Analysis (SI, Chapter 6) of the VOIs obtained from the outer edge of the sample confirms that they are formed by almost perfect spherical grains (mean grain sphericity of 1.0 ± 0.1). The increase of the connectivity density and the higher SC vol% close to the bottom layer of the sample relates to the fact that same grain sizes, but with a much higher interconnectivity between them is found close to the lower part of the nanocomposite.

For the evaluation of the top region of the sample (in green in Fig. 5A,E), 3 VOIs were selected, all at *approx*. 1 mm from the bottom of the sample, but at increasing distances from the edge of the sample (Fig. SI12). Again, the Blob Analysis (SI, Chapter 6) of the VOIs obtained confirms that this region is also constituted by almost perfect spherical grains (mean grain sphericity of 1.0 ± 0.1). Nevertheless, in this case, a strong dependency of the microstructure with the VOI position exists. Highly interconnected SC grains are found in the inner part of the samples, whereas slightly smaller but more interconnected supercrystalline grains are formed in the inner part. The opposite trend is observed for the vol% of the supercrystalline phase, which increases with the edge-distance.

The last selected region corresponds to the inner part of the sample, at *approx*. 0.5 mm above the bottom of the sample (in orange in Fig. 5A,D). Very small highly inter-connected supercrystalline grains form this region. These grains are so closely interconnected that single spherical grains cannot be identified, hampering a reliable quantification of the mean grain size.

Such an analysis of the various regions appearing in Batch 2 nanocomposites offer insights on the mechanisms governing the formation and distribution of the different phases, as described in the Discussion section.

### Mechanical properties of the nanocomposite materials

The mechanical properties of the two types of nanocomposites (Batch 1 and Batch 2) were assessed *via* nanoindentation. Because of the existence of macroscopic cracks, methods like Vickers-indentation, which probe a larger volume, were not applied. A set 200 nm-deep Berkovich tip nanoindents over an area of 600 × 600 µm^2^ was performed (see Methods section). The 200 nm depth was selected to ensure that the indents’ areas were smaller than the average size of the Batch 2 supercrystalline grains, as resolved via SRµCT, and thus to decouple the mechanical behavior of the two phases characterizing the material. For Batch 2 material, and in order to have a representative evaluation of the mechanical behavior of the hierarchical structure, the evaluation was performed in an area with spherical supercrystalline grains. For Batch 1, a polished fracture surface was evaluated.

**Figure 6:**
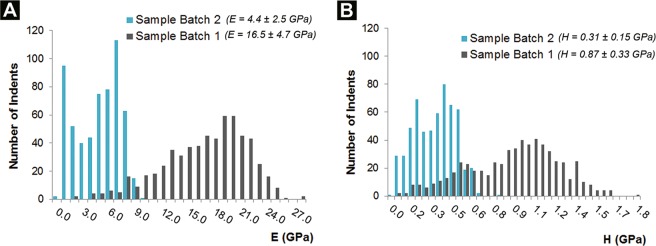
Distribution of the elastic modulus (**A**) and hardness (**B**) of the materials obtained with Batch 1 and Batch 2 showing a remarkably different mechanical behavior, depending on the amount of organic phase used for the preparation of the nanocomposite materials.

Fig. 6 shows the obtained values of elastic modulus (*E*) and hardness (*H*) for the two materials, together with their distributions. Several interesting features emerge, confirming how the two different microstructures induce a distinct behavior in the nanocomposites. The two most noticeable aspects are the overall lower values and the bimodal distributions characterizing the mechanical properties of the nanocomposites obtained with Batch 2. The bimodal distribution, in particular, confirms the expected behavior of such a hierarchically-structured material, made of ceramic-rich supercrystalline grains surrounded by an organic-rich matrix (see Fig. 2). The highest values of hardness and elastic modulus obtained with Batch 2 (in the ranges of 0.4–0.8 GPa and 7–10 GPa, respectively) are attributed to the contribution of the supercrystalline grains, while the values at the lowest ends (*H* between 0.1–0.3 GPa and *E* of 0.1–2 GPa) are attributed to the surrounding OPh-rich phase. The intermediate values can be ascribed to the presence of areas with very small supercrystalline grains (see SRµCT results displayed in the previous section), in which the indents’ extension is such that the contributions of both, supercrystalline and organic phase, play a role in the mechanical properties measurements. Even in areas characterized by larger supercrystals (several µm-sized), the position of the single indents within these grains also determines the measured modulus and hardness: the closer to the grains’ boundaries the measurement is performed, the higher the influence of the weaker surrounding matrix is.

However, the other feature of Fig. 6 mentioned above – overall higher values of modulus and hardness for Batch 1 with respect to Batch 2 – suggests that the influence of the organic-rich phase in Batch 2 is affected also in the case of indentation tests performed in central positions of the SC grains. Indeed, the highest values of hardness and modulus obtained for Batch 2 (up to 0.76 GPa and 9.3 GPa, respectively) are still less than half of the highest ones related to Batch 1 (1.76 and 27.9 GPa). The combination of an indentation area such that the zone of interaction of each indent with the surrounding material is larger than most supercrystalline grains, together with the softness of the surrounding OPh-based matrix, leads to the global decrease of mechanical properties in the organic-rich nanocomposites.

## Discussion

The common microstructural feature of the samples from Batch 1 and Batch 2 is that they have SC-grains as a major constituent (Level 1). In both batches the determined interparticle distances (IDs) of *approx*. 1 nm are much smaller than the length of the extended oleyl phosphate (OPh) molecule, therefore in the FCC superlattices obtained the OPh molecules are interdigitated or bent. Moreover, depending on the weight fraction of OPh in the starting material, small differences in the ID are observed. When the material is obtained from a low content OPh suspension, slightly smaller IDs are found, while when the material is obtained with the high organic content suspension, a larger distance between NPs is observed. This is in agreement with the results of the elemental analysis revealing different amounts and species of OPh for the two batches. In the case of the low organic content, mainly the monoester remains attached to the NPs, whereas for the high organic content, a mixture of mono- and di-ester is present. Additionally an excess of organic ligand can be trapped in-between the interstitial sites of the supercrystals when NPs self-assemble, thus affecting the packing during the self-assembly, and also form bilayers, thereby increasing the distance between the NPs’ cores^19,30^.

The results presented in Figs. 3–5 show that the sample of Batch 2 is very inhomogeneous with a graded structure. These results also confirm that the supercrystalline grains are mainly composed by the organo-modified iron oxide nanoparticles, while the phase in-between them is mainly organic material - the excess of OPh in the starting suspension. Therefore, the hierarchical structure formation obtained with Batch 2 can be understood as a phase separation between OPh (in excess) and the OPh-functionalized Fe_3_O_4_-nanoparticles (Fe_3_O_4_-OPh-NPs). As the supercrystals are an own phase with an almost stoichiometric ratio between OPh and Fe_3_O_4_, corresponding to 12 wt% OPh (as shown by elemental analysis and SAXS), any further addition of OPh leads to a phase separation when the suspension (a mixture of Fe_3_O_4_-OPh-NPs, OPh, and toluene) is dried (toluene evaporates). This leads to the development of a hierarchical two-phase nanocomposite material consisting of supercrystalline grains of Fe_3_O_4_-OPh-NPs surrounded by the excess of OPh (Fig. 4).

Moreover, based on the microstructural analysis by SRµCT, the complex 3D structure of the nanocomposite can be related to the mechanism of the self-assembly process itself. First of all, besides the bottom layer of the sample of Batch 2, only almost perfect spherical SC grains are identified (Fig. 5). Hence, we assume that, as in a classical crystallization mechanism, the Fe_3_O_4_-OPh-NPs self-assemble into supercrystals by nucleation and growth in the suspension when a critical concentration of functionalized NPs is reached^15^. As the SC are almost perfectly spherical it can be concluded that the growth velocity is identical in all directions. Also, it is assumed that the self-assembly starts at the suspension-air interface^31,32^, and it is also being promoted at the walls of the vessel (suspension-wall interface). This can be inferred from the SRµCT results, which show a clear increase of the size of the SC grains at the outer edge of the sample (Fig. 5C). Furthermore, since *approx*. 12 wt% OPh corresponds to a monolayer of OPh on the NPs’ surface and this is the amount present in the supercrystalline grains of both batches, the rest of OPh in Batch 2 is most probably in the form of free molecules in the colloidal suspension. Thus, the starting suspension in Batch 2 can be considered as a mixture of 3 species, which are the Fe_3_O_4_-OPh-NPs, the OPh free molecules, and the toluene. As the evaporation of toluene proceeds, the average distance of the Fe_3_O_4_-OPh-NPs in the suspension decreases until a certain Fe_3_O_4_-OPh-NPs concentration, after which the self-assembly starts and proceeds with a radially-symmetrical SC growth. As these NPs self-assemble to form supercrystalline grains that become progressively heavier, their surface to volume ratio decreases, as well as the ratio of viscous drag force to the gravitational force, described quantitatively by Stoke’s law^33^. As a consequence, the bigger the grains become, the faster they move towards the bottom of the vessel. At the bottom of the vessel they partly merge to even bigger supercrystals, forming the flat supercrystalline layer observed at the bottom, with some grain boundaries remaining between the supercrystals, as can be clearly seen in the Supplementary video and in Fig. 5. This explanation is also in agreement with the fact that close to the bottom of the sample the connectivity between the grains is higher, an observation that can be understood as a result of a grain coarsening process. While these supercrystals form a bottom flat supercrystalline layer, and given that in the supercrystals there is only *ca*. 12 wt % OPh, the ratio OPh to Fe_3_O_4_-OPh-NPs in the remaining suspension increases (the rest up to the *approx*. 32 wt% - original composition of Batch 2 - remains in suspension). Thus, since the concentration of OPh in toluene is much higher than at the start of the self-assembly process, the growth of the supercrystalline grains is affected. In addition, as the remaining suspension is enriched with OPh, which has a much higher viscosity than toluene, the velocity of the NPs moving towards each other and downward towards the bottom is much smaller than in the first case. This means that supercrystals start to nucleate, but, as there is such abundancy of organic phase in-between the formed supercrystalline nuclei, their growth and further coarsening is partly impeded. Thus, smaller supercrystalline grains are obtained. Finally, at the very last stage of the self-assembly process, the toluene evaporates completely and a mixture of supercrystals with OPh is left. The size and distribution of the supercrystalline grains at this point will mainly depend on the particle growth rate, the evaporation rate and the viscosity of the suspension.

In summary the complex hierarchical microstructure of the sample from Batch 2 results from an interplay of nucleation and growth, gravitational sedimentation, and merging of SC grains.

The nanoindentation experiments performed in this study show higher values of elastic modulus and hardness of purely supercrystalline materials (Batch 1), which is due to the tight packing of their constituent nanoparticles (packing factor of 0.74 for FCC). The large scatter that is nevertheless affecting the mostly uniformly supercrystalline material obtained with Batch 1 is a well-known issue of these kinds of self-assembled nanocomposites^10,34^, and can be due to several factors, ranging from the presence of defects at the superlattice level and areas with localized excess of organic material, to varying orientations of the superlattice with respect to the applied load. The materials obtained with Batch 2, on the other hand, show less broadening of the scatter data, partly due to the lower overall properties achieved, but also due to the different behavior of supercrystalline grains and matrix. The left-tailed shape of the Batch 1 histogram is likely due to the organic material distribution, which is affected by inhomogeneities induced at the end of the self-assembly process, when the material remaining in suspension has very limited chances for rearrangement and optimal self-organization. The slightly bimodal distribution that seems to appear in the hardness data of the same Batch 1 can also be due to the presence of these areas containing excess of organic material, and thus with less tightly packed ceramic NPs. Chipping and cracking phenomena are also likely to arise here.

Comparing with previous work conducted with a similar material system, magnetite-oleic acid nanocomposites with analogous iron oxide NPs and organic content as in Batch 1, the measured values of hardness and elastic modulus are lower in the oleyl phosphate case (*E* = 16.5 ± 4.7 GPa and *H* = 0.87 ± 0.33 GPa for Batch 1 of Fe_3_O_4_-OPh compared to *E* = 34.6 ± 2.2 GPa and *H* = 1.39 ± 0.17 GPa for Fe_3_O_4_-oleic acid)^10^. Such a difference can be due to the fact that the oleyl phosphate’s anchoring group contains an ester bond, which is known to be weaker than the C-C aliphatic bond^35^. Additionally, the two materials were produced starting from distinct initial NPs’ batches, and the nanoindentation measurements were conducted with slightly different parameters.

Based on structural biomaterials such as nacre, five main requirements can be defined in order to obtain synthetic materials with improved mechanical properties - hard, strong, and damage tolerant-: i) a hierarchical structure, ii) an anisotropic microstructure (i.e. with aligned platelets or needles surrounded by a weak matrix phase), iii) a low % in volume of the soft phase, iv) a strong difference in elastic modulus between the hard and the soft phase^1,6^, and v) an optimal combination of strength and aspect ratio of the first hierarchical level bricks and the strength of the soft matrix for the second hierarchical level. Thus, although the hierarchical SC structure based on spherical grains seems not to be the most adequate for the purpose, a further control in order to tune the NPs self-assembly is a promising next step to tune the geometry and properties of tailored nanocomposites. In this regard it is also important to note that the flat platelet-like grains appearing at the SC bottom layer of Batch 2 samples (as shown in Figs 4, 5B) already point in the right direction. By controlling the aspect ratio of such a platelet-like SC material and stacking the platelets while adjusting the amount of organic phase would allow obtaining a final hierarchical macroscopic material that fulfils the main requirements before-stated.

## Conclusions

For the first time, it has been shown that by self-assembly *via* solvent evaporation it is possible to tune the microstructure of bulk macro-sized nanocomposites and directly synthesize a hierarchical structure. The formation of such a hierarchical structure is related to the self-assembly mechanism, and the gained knowledge can be extrapolated to other similar nanoparticle systems with ligands that remain in a condensed state at room temperature.

Moreover, while bulk nanocomposites with uniform supercrystallinity still show a very high potential for the achievement of enhanced hardness, stiffness, and strength, organic-rich nanocomposites with hierarchical microstructures offer room for tailoring their mechanical behavior, and possibly provide higher fracture toughness.

## Methods

### Preparation of oleyl-phosphate modified iron-oxide nanoparticles

A solution of oleic acid stabilized iron oxide nanoparticles (12 g from CAN GmbH, Germany, Fig. SI1) in toluene was added under stirring to an oleyl phosphate mixture (40/60% (w/w) mono-/di-ester, 25 g) in toluene. The solution was further stirred at room temperature for 48 h. Afterwards, the particles were precipitated by adding an excess of acetone (1/1.5 (v/v)) and separated from the supernatant by centrifugation (8000 × g, 10 min). The resulting sediment was washed twice with 200 mL acetone followed by several washing steps with 2-propanol (200 mL/washing step). To efficiently remove the excess of ligand during these washing steps, the samples were placed into an ultrasonic bath (5 min). After washing, the particles were re-dispersed in toluene or chloroform (700 mL). The procedure of precipitation, washing and re-dispersing was repeated, until the desired organic content was set.

### Characterization of the oleyl-phosphate modified iron-oxide nanoparticles

Thermogravimetric Analysis (TGA) during the washing steps was performed using a Netzsch TGA 209 F1 Iris, from 25 to 800 °C at 5 °C/min under N_2_ flux (60 mL/min.) TGA of the starting suspensions and pellets of the self-assembled material were carried out under N_2_ flux (820 mL/min) at 1 °C/min from 25 to 900 °C using a Mettler Toledo TGA/DSC 1 STARe System. Attenuated Total Reflectance-Fourier Transform Infrared Spectroscopy (ATR-FTIR) was performed on a Varian 660 FTIR spectrometer (Agilent, United States) equipped with a Pike MIRacle single reflection ATR system (Pike Technologies, United States). Solutions of the samples were dried on the crystal and the resulting films were measured with a resolution of 4 cm^−1^ by averaging 64 scans. Elemental analysis of nanoparticle powder samples for carbon and hydrogen was performed on a EuroEA3000 Elemental Analyzer (Eurovector, Italy). For the analysis of iron and phosphorous, the samples were pre-treated with nitric and perchloric acid for chemical pulping. The resulting solutions were analysed by Inductively Coupled Plasma-Atomic Emission Spectroscopy (ICP-AES) on a SPECTRO ARCOS system (SPECTRO Analytical Instruments GmbH, Germany). The starting nanoparticles were evaluated using Transmission Electron Microscope (TEM) JEOL JEM-1011 (Jeol, USA) at 100 kV by depositing a drop of the corresponding diluted suspension on a carbon coated 400 mesh TEM grid. Small Angle X-Ray Scattering (SAXS) measurements were performed at the high energy materials science (HEMS) beamline^36^, at the PETRA III storage ring at the Deutsches Elektronen-Synchrotron (DESY). The energy of the incident beam was 87.1 keV (wavelength: 0.01423 nm) and had a cross section of 0.2 × 0.2 mm^2^. A two-dimensional PerkinElmer detector with a pixel size of 200 µm was placed at a sample-to-detector distance of 3400 mm to detect the scattering signal. For the calculation of the particle sizes the software Scatter was used, assuming a hard sphere model and a log-normal size distribution of the radius^37^.

### Preparation of the supercrystalline nanocomposite materials

Starting suspensions of oleyl phosphate-coated iron oxide nanoparticles in toluene (130 g/L) were self-assembled by slow evaporation of the solvent carrier within several days at room temperature in a cylindrical vessel. Thereafter, in order to obtain a better handling of the material, the remaining sediments were dried under vacuum at room temperature for 30 min and subsequently pressed via uniaxial pressing at 150 °C. The loads applied in this step were 51.0 MPa and 95.5 MPa, for Batch 1 and Batch 2 samples respectively. Note that the temperature of this step was selected in order to obtain the most suitable rheology of the organic phase for the pressing step, but also to be low enough to ensure that the ligand did not decompose (Fig. SI3).

### Characterization of the supercrystalline nanocomposite materials

Scanning Electron (SE)-images were taken with a Zeiss Supra VP55 (Zeiss, Germany) at 1.5 kV, 10 µm aperture size, in high vacuum mode, and using the ETD detector. Specimens were mounted on a Scanning Electron Microscope (SEM) sample holder using Silver glue (Acheson Silver DAG 1415 M). SEM- Energy-Dispersive X-ray Spectroscopy (EDX) measurements were performed at 20 kV and 0.8 nA on a FEI Helios G3 UC (FEI, USA) equipped with an Oxford X-MAX 80 mm² SDD. The samples were coated with gold prior to the EDX measurements. A FIB lamella for TEM-EDX was prepared using a standard liftout procedure using the above-mentioned FEI Helios G3 (FEI, USA) system. As a support grid, an EMtec copper liftout grid was used. Final thickness of the lamella is estimated to be around 60 nm. TEM-EDX was performed at 200 kV and 1 nA on a FEI Talos F200X (FEI, USA) equipped with a four quadrant Super-X EDX System. Spectrum image size: 256 × 256 pixels. Pixel-size: 765 pm. Dispersion: 5 eV. Dwell-time: 5 µs. Overall measuring time: 60 minutes with drift compensation. For the characterization of the supercrystals, obtained SAXS measurements were performed by scanning a line along the samples and using the same parameters and post-processing as stated before.

### Characterization by Synchrotron Micro-Tomography (SRµCT)

The sample was scanned at the P07 beamline for High Energy Materials Science at the Petra III storage ring at DESY^36^. The scanning parameters were as follows, energy: 45.4 keV, sample rotation: 180 degrees, effective pixel size: 1.32 µm, number of projections: 1200, angular step: 0.15 degrees, FOV (Field of View): 3.1 × 2.4 mm^2^, binning 1 × 1, format: 16-bit. For the post processing, the x-ray linear attenuation coefficient, µ, (cm^−1^) was reconstructed on a 32-bit grey level scale. In order to remove image noise, a 3D mean filter (size: 3 × 3 × 3 pixels^3^)^38^ implemented in Image J (US National Institutes of Health, Bethesda, MD, USA). Since the quantitative analysis software package further described below only supports 8-bit or 12-bit images, the reconstructed slices were then converted to an 8-bit grey scale range. The down-conversion to 8-bit was performed using Image J and no loss of information in the images was observed. Hereinafter the low- and high-absorbing parts of the sample were separated using standard grey-level thresholding based on Otsu’s method^39^. Quantitative analysis was performed on a set of VOIs (Volumes of Interests) placed out in different sample-specific regions of the sample using the Pore3d software library^40^. The following quantitative parameters were computed: percentage volume (%vol.), mean grain size (µm), sphericity, and connectivity density (mm^−3^). Isolated skeleton branches, which were not connected to the longest skeleton, were filtered out. The image skeleton was computed using the Gradient Vector Flow (GVF) algorithm^41^ and implemented in Pore3d using the following parameters: scale = 1.00, hierarchy = 0.40 and connectivity = 26. The representative sizes of these VOIs were selected via a Representative Volume of Interest test (RVI-test)^42^. Herein-after the quantified parameters were averaged with respect to the distance from either the bottom or the perimeter of the sample. More information is available at SI, Chapter 6.

### Nanomechanical testing

Samples were evaluated by Nanoindentation. Before the nanoindentation tests, a portion of each sample (area of ca. 2 500 mm^2^) was embedded in a cold curing acrylic mounting resin, and polished down to a roughness of 50 nm using a sequence of SiC papers and diamond suspensions (for 15–0.25 µm from ATM GMBH, Germany, and for 0.05 µm from Buehler, Germany). The mechanical tests were performed in an Agilent Nano Indenter G200 (Agilent, USA) system using the CSM (continuous stiffness measurement) method with a constant strain target of 0.05 s^−1^, a harmonic displacement target of 2 nm and a harmonic frequency of 45 Hz. The maximum indentation depth was set to 200 nm. The system was equipped with a Berkovich tip. The number of indents per sample was 625, arranged in a square of 25 by 25 indents covering an overall area of 0.36 mm^2^. The nanoindenter conducted the measurements in displacement-control mode. After the desired indentation depth was reached, the load was held constant for 10 seconds before withdrawing the tip from the sample. The area of each indent was calculated based on literature^43^ using a contact depth of 110 nm, leading to an estimated imprint area of 0.30 µm^2^ and a lateral size of 0.83 µm per indent.

## Supplementary information

Supplementary information

Supplementary video

## References

[1] Munch E (2008). Tough, bio-inspired hybrid materials. Science.

[2] Ji B, Gao H (2010). Mechanical Principles of Biological Nanocomposites. Annu. Rev. Mater. Sci..

[3] Meyers MA, Chen P-Y, Lin AY-M, Seki Y (2008). Biological materials. Structure and mechanical properties. Prog. Mater Sci..

[4] Gao H, Ji B, Jäger IL, Arzt E, Fratzl P (2003). Materials become insensitive to flaws at nanoscale. Lessons from nature. Proc. Natl. Acad. Sci..

[5] Rajasekharan AK, Bordes R, Sandstrom C, Ekh M, Andersson M (2017). Hierarchical and Heterogeneous Bioinspired Composites-Merging Molecular Self-Assembly with Additive Manufacturing. Small.

[6] Wegst UGK, Bai H, Saiz E, Tomsia AP, Ritchie RO (2015). Bioinspired structural materials. Nat. Mater..

[7] Mout R (2017). Programmed Self-Assembly of Hierarchical Nanostructures through Protein-Nanoparticle Coengineering. ACS nano.

[8] Zhang W, Liao SS, Cui FZ (2003). Hierarchical Self-Assembly of Nano-Fibrils in Mineralized Collagen. Chem. Mater..

[9] Fan H (2018). Formation of self-assembled gold nanoparticle supercrystals with facet-dependent surface plasmonic coupling. Nat. Commun..

[10] Dreyer A (2016). Organically linked iron oxide nanoparticle supercrystals with exceptional isotropic mechanical properties. Nat. Mater..

[11] Qin X, Luo D, Xue Z, Song Q, Wang T (2018). Self-Assembled Ag-MXA Superclusters with Structure-Dependent Mechanical Properties. Adv. Mater..

[12] Zhang H (2017). Assembling and ordering polymer-grafted nanoparticles in three dimensions. Nanoscale.

[13] Wright A (2006). Hierarchically Organized Nanoparticle Mesostructure Arrays Formed through Hydrothermal Self-Assembly. Chem. Mater..

[14] Liu Q, Sun Z, Dou Y, Kim JH, Dou SX (2015). Two-step self-assembly of hierarchically-ordered nanostructures. J. Mater. Chem. A.

[15] Wang C, Siu C, Zhang J, Fang J (2015). Understanding the forces acting in self-assembly and the implications for constructing three-dimensional (3D) supercrystals. Nano Res..

[16] Reichhelm A, Haubold D, Eychmüller A (2017). Ligand Versatility in Supercrystal Formation. Adv. Funct. Mater..

[17] Si KJ, Chen Y, Shi Q, Cheng W (2018). Nanoparticle Superlattices: The Roles of Soft Ligands. Adv. Sci..

[18] Boles MA, Engel M, Talapin DV (2016). Self-Assembly of Colloidal Nanocrystals. From Intricate Structures to Functional Materials. Chem. Rev..

[19] Bian K, Li R, Fan H (2018). Controlled Self-Assembly and Tuning of Large PbS Nanoparticle Supercrystals. Chem. Mater..

[20] Sturm EV, Colfen H (2016). Mesocrystals. Structural and morphogenetic aspects. Chem. Soc. Rev..

[21] Weidman MC, Nguyen Q, Smilgies D-M, Tisdale WA (2018). Impact of Size Dispersity, Ligand Coverage, and Ligand Length on the Structure of PbS Nanocrystal Superlattices. Chem. Mater..

[22] Borchert H (2005). Determination of Nanocrystal Sizes. A Comparison of TEM, SAXS, and XRD Studies of Highly Monodisperse CoPt3 Particles. Langmuir.

[23] Sarmphim P (2018). FePt3 nanosuspension synthesized from different precursors – a morphological comparison by SAXS, DLS and TEM. B. Pol. Acad. Sci-Tech..

[24] Daou TJ (2007). Phosphate Adsorption Properties of Magnetite-Based Nanoparticles. Chem. Mater..

[25] Bixner O, Lassenberger A, Baurecht D, Reimhult E (2015). Complete Exchange of the Hydrophobic Dispersant Shell on Monodisperse Superparamagnetic Iron Oxide Nanoparticles. Langmuir.

[26] Roonasi P, Holmgren A (2009). A Fourier transform infrared (FTIR) and thermogravimetric analysis (TGA) study of oleate adsorbed on magnetite nano-particle surface. Appl. Surf. Sci..

[27] Rudolph M, Erler J, Peuker A (2012). U. A. TGA-FTIR Perspective of Fatty Acid Adsorbed on Magnetite Nanoparticles - Decomposition Steps and Magnetite Reduction. Colloid. Surface. A.

[28] Goldstein, J. I. *et al*. *Scanning electron microscopy and* X-ray *microanalysis* (ed. Joy, D.C., Echlin, P., Goldstein, J.I.) (Springer, 2017).

[29] Cliff G, Lorimer GW (1975). The quantitative analysis of thin specimens. J. Microsc..

[30] Lee SS (2015). Engineered manganese oxide nanocrystals for enhanced uranyl sorption and separation. Environ.Sci.-Nano.

[31] Josten E (2017). Superlattice growth and rearrangement during evaporation-induced nanoparticle self-assembly. Sci. Rep..

[32] Geuchies JJ (2016). *In situ* study of the formation mechanism of two-dimensional superlattices from PbSe nanocrystals. Nat. Mater..

[33] *A Dictionary of Physics* (ed. Law, J. & Rennie, R.) (Oxford University Press, 2015).

[34] Tam E (2010). Mechanical Properties of Face-Centered Cubic Supercrystals of Nanocrystals. Nano Lett..

[35] Blanksby SJ, Ellison GB (2003). Bond Dissociation Energies of Organic Molecules. Accounts Chem. Res..

[36] Schell N (2014). The High Energy Materials Science Beamline (HEMS) at PETRA III. Mater. Sci. Forum.

[37] Forster S, Apostol L, Bras W (2010). Scatter: Software for the analysis of nano- and mesoscale small-angle scattering. J. Appl. Cryst..

[38] Ollion J, Cochennec J, Loll F, Escudé C, Boudier T (2013). TANGO: A generic tool for high-throughput 3D image analysis for studying nuclear organization. Bioinformatics.

[39] Otsu. N, Threshold A (1979). Selection Method from Gray-Level Histograms. IEEE Transactions on Systems, Man, and Cybernetics.

[40] Brun F (2010). Pore3D. A software library for quantitative analysis of porous media. Nucl. Instrum. Methods Phys. Res..

[41] Brun F, Dreossi D (2010). Efficient curve-skeleton computation for the analysis of biomedical 3d images. Biomed. Sci. Instrum..

[42] Bear, J. *Dynamics of fluids in porous media* (Courier Corporation, 1988).

[43] Oliver WC, Pharr GM (1992). An improved technique for determining hardness and elastic modulus using load and displacement sensing indentation experiments. J. Mater. Sci..

