# Supplementary material for: Hierarchical supercrystalline nanocomposites through the self-assembly of organically-modified ceramic nanoparticles
Source: Sci Rep. 2019 Mar 5;9:3435. doi: 10.1038/s41598-019-39934-4 (PMC6401156; doi:10.1038/s41598-019-39934-4)
Supplement: Supplementary file 1 — Supplementary information [file 41598_2019_39934_MOESM1_ESM.docx]

Supporting Information

Hierarchical supercrystalline nanocomposites through the self-assembly of organically-modified ceramic nanoparticles

Berta Domènech*, Michael Kampferbeck, Emanuel Larsson, Tobias Krekeler, Büsra Bor, Diletta Giuntini, Malte Blankenburg, Martin Ritter, Martin Müller, Tobias Vossmeyer, Horst Weller, Gerold A. Schneider*

1. **Nanoparticle preparation and characterization.**


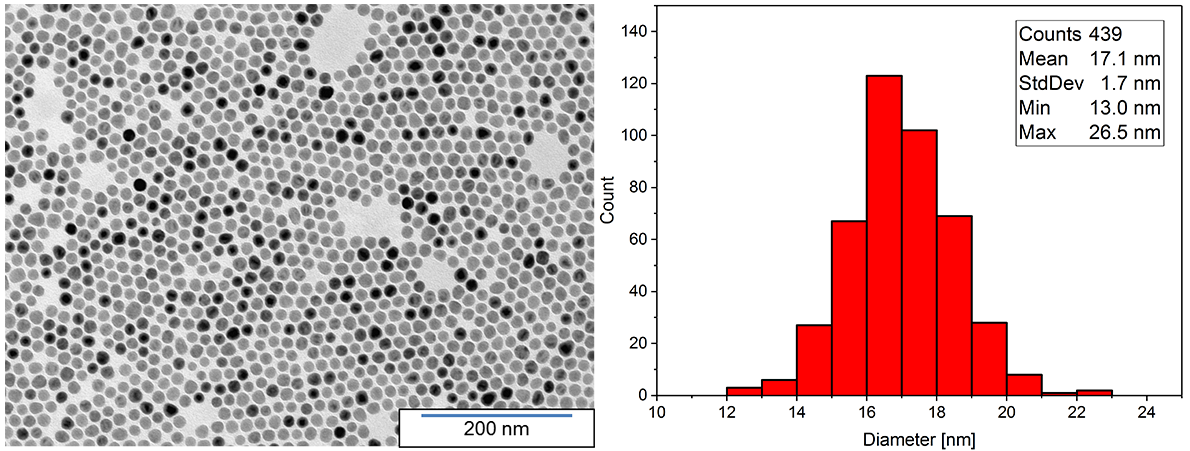


**Figure SI 1.** TEM image of iron oxide nanoparticles obtained from CAN GmbH with the corresponding size-distribution histogram.

Comparing the TEM and SAXS results for the nanoparticles (NPs) diameter after the ligand exchange and washing procedure (Figure 1C and 1D in the main article) with those obtained with the original oleic acid stabilized particles here presented) it is clear that neither the ligand exchange nor the washing procedure had an effect on the particle sizes and their morphology. The small size differences obtained within these two values may be due to an error in the diameter determination associated to the methodology used.^[^^1]^

**
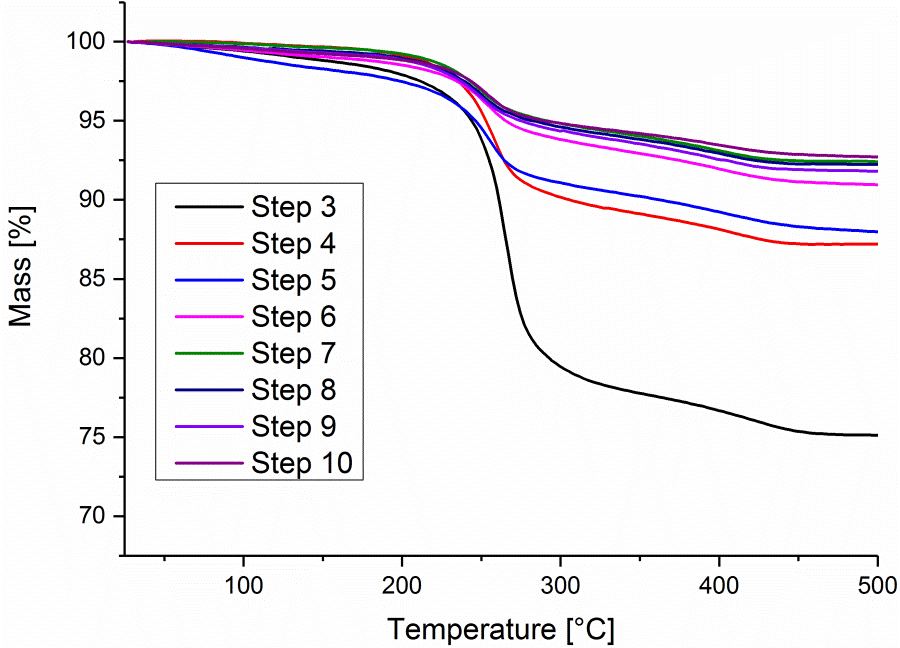
**

**Figure SI 2.** TGA curves of the Fe_3_O_4_-OPh NPs after each of the washing steps showing a reduction of the organic content (measured between 150 – 500 °C) when increasing the number of washing steps.


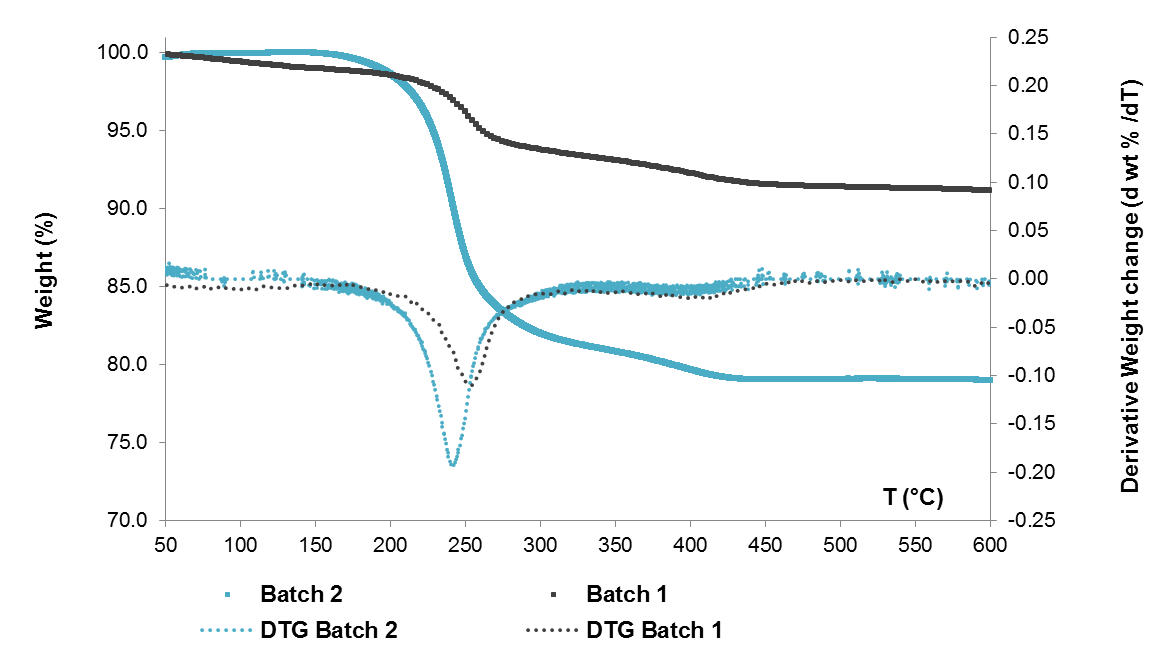


**Figure SI 3.** TGA and the corresponding first derivative (DTG) of the starting suspensions (Batch 1 and Batch 2). In both cases it can be noted that there are two distinct transitions between 50 °C and 600 °C, being the first weight loss (DTG peak at around 250 °C) much more sharply pronounced than the second weight loss (DTG peak at ca. 400 °C). Sahoo *et al.*^[^^2]^ proposed a quasi-two-layered adsorption model of phosphate molecules on magnetite nanoparticles, wherein the two peaks could be understood as a stepwise desorption, due to a differential bonding of the organic molecules to the nanoparticle’s core. In contrast, FTIR-TGA studies on magnetite nanoparticles covered with oleic acid and other fatty acids show that the steps are attributed to a number of decomposition and desorption processes of the ligands at the nanoparticle surface, including decomposition under the release of CO_2_ and dehydrogenation.^[^^3,4]^ Nevertheless, just by the TGA data it is not possible to discern if the total organic is in the form of a monolayer on the nanoparticles surface or if there is a bi-layer absorption.


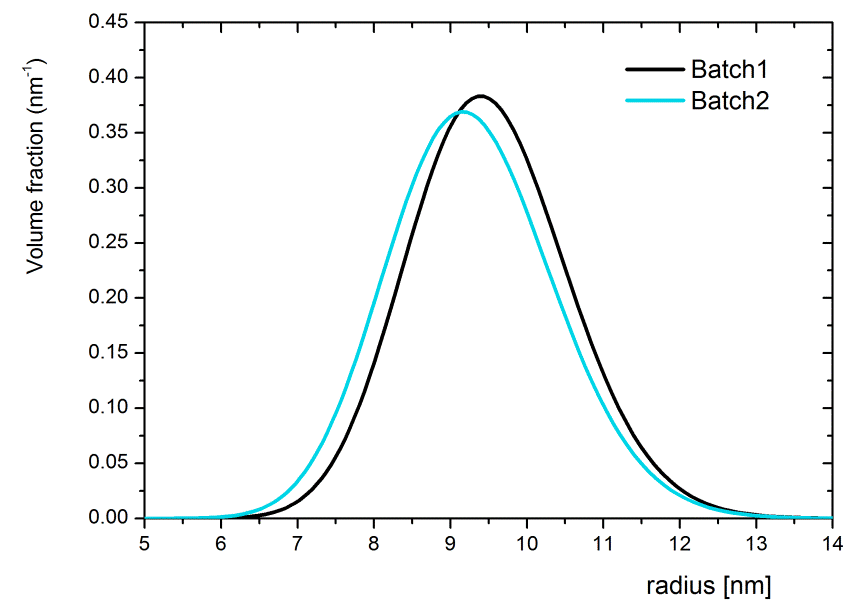


**Figure SI 4.** The log-normal size distributions of the radius of the initial suspensions measured with SAXS.

**
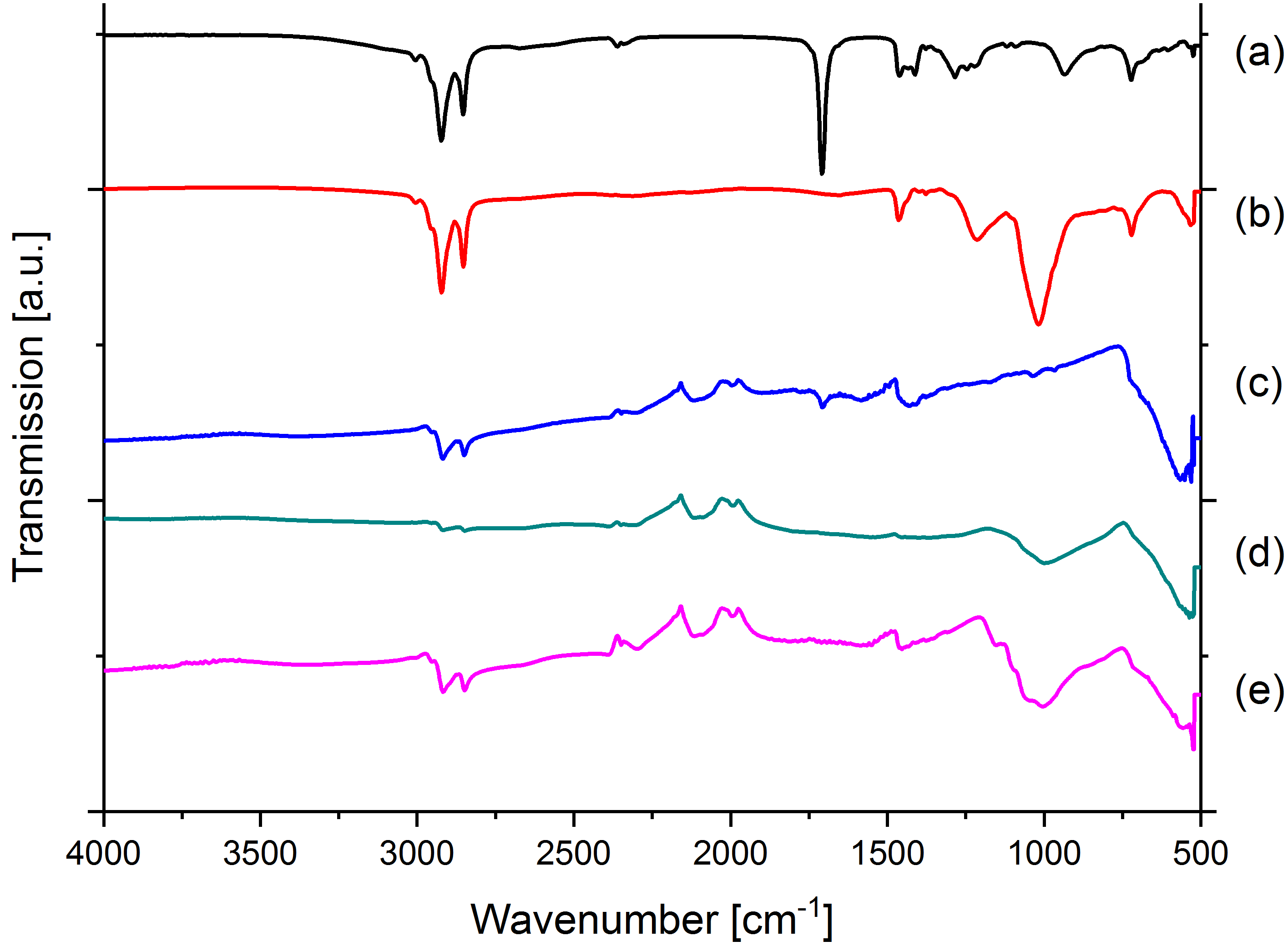
**

**Figure SI 5.**  FTIR Spectra of Oleic acid (a), Oleyl phosphate (b), Fe_3_O_4_-OA NPs(c), Fe_3_O_4_-OPh 8 wt% suspension, Fe_3_O_4_-OPh 21 wt% suspension. Fe_3_O_4_-OPh samples show four strong bands between 1250 and 900 cm^-1^ (located at 1152 cm^-1^, 1095 cm^-1^, 1050 cm^-1^, and 1000 cm^-1^), which are attributed to adsorbed phosphate on an iron oxide surface Free oleyl phosphate shows two strong bands in this region for P=O (1213 cm^-1^) and P-O-C (1016 cm^-1^) stretching vibrations, whereas oleic acid coated NPs only show two very faint bands at 1040 and 966 cm^-1^. The first band is attributed to C-O stretching vibration, the second representing the out-of-plane bending vibration of residual unbound dimeric carboxylic acid.

1. **Grafting Density obtained from Elemental Analysis of the Starting iron oxide – oleyl phosphate suspensions.**

Table SI 1. Results for both batches obtained from Elemental Analysis. All values are expressed in weight %.

|  | Batch 1 | Batch 2 |
| --- | --- | --- |
| Fe | 63.79 | 50.77 |
| P | 1.11 | 2.39 |
| C | 7.81 | 21.64 |
| H | 1.28 | 3.51 |
| O (calculated) | 26.01 | 21.69 |

With a fictive sample weight of *m*_ges_ = 1 g and the obtained values in weight% *w* from elemental analysis, the number of atoms *N* is calculated.

|  | $N=n\cdot N_{A}=\frac{m}{M}\cdot N_{A}=\frac{w\cdot m_{\text{ges}}}{100 M}\cdot N_{A}$ | (1) |
| --- | --- | --- |

For magnetite, the volume of the unit cell is given as *V*_UC_ = 591.46 Å^3^ with 8 formula units and therefore 24 iron atoms per cell. With this and the particle diameter *d* obtained from SAXS, the number of iron atoms per particle can be calculated, assuming perfect spheres:

|  | $N_{\text{Fe, particle}}=24\cdot\frac{V_{\text{particle}}}{V_{\text{UC}}}=\frac{4\pi d^{3}}{V_{\text{UC}}}$ | (2) |
| --- | --- | --- |

The number of particles *N*_particles_ is calculated from the ratio of the absolute number of iron atoms *N*_Fe_ and the number of iron atoms per particle *N*_Fe, particle_. From the number of particles, the overall surface of the sample (all magnetite nanoparticles) *S*_Sample_ is calculated:

|  | $S_{\text{Sample}}=N_{\text{particles}}\cdot\pi d^{2}=\frac{N_{\text{Fe}}}{N_{\text{Fe, particle}}}\cdot\pi d^{2}$ | (3) |
| --- | --- | --- |

As the number of phosphorous atoms *N*_P_ equals the number of phosphate binding groups, the number of phosphorous atoms is used to calculate the grafting density of the phosphate:

|  | $\sigma_{\text{Phosphate}}=\frac{N_{\text{P}}}{S_{\text{Sample}}}$ | (4) |
| --- | --- | --- |

With equations 1-3, the calculation can be simplified to equation 5:

|  | $\sigma_{\text{Phosphate}}=\frac{w_{\text{P}}}{M_{\text{P}}}\cdot\frac{M_{\text{Fe}}}{w_{\text{Fe}}}\cdot\frac{4d}{V_{\text{UC}}}$ | (5) |
| --- | --- | --- |

With C/P-ratio, the grafting density of aliphatic chains can be calculated:

|  | $\sigma_{\text{Chains}}=\sigma_{\text{Phosphate}}\cdot\frac{C/P}{18}$ | (6) |
| --- | --- | --- |

For Batch 1 a phosphorous content of 1.11 wt% was obtained. With the measured iron content of 63.79 wt% and the determined by SAXS diameter of 18.4 nm (and assuming a perfect spherical shape), this phosphorous content translates into a grafting density of phosphate onto the iron oxide surface of 3.9 molecules/nm^2^. The total organic content for this component was calculated by taking into account the sum of all elements of the ligands, resulting in a mass fraction of 12.4 wt%. Because the measured C/P-ratio is 18.3/1.0, it can be stated that, from the original mixture of mono- and diester OPh used for the ligand exchange reaction, the monoester is the only one remaining on the surface of the iron oxide. Thus, it can be concluded that the diester was removed almost completely during the washing procedure. Furthermore, the ratio C/P found by the elemental analysis also confirms the ATR-FTIR measurements, suggesting that the oleic acid ligands were exchanged by OPh monoester nearly quantitatively. In addition, it is noted that the measured hydrogen content is in agreement with the sum formula of the oleic residue of oleyl phosphate.

For Batch 2, a phosphorous content of 2.39 wt% was obtained. With the measured iron content of 50.77 wt%, a theoretical grafting density of 10.7 molecules/nm^2^ was calculated suggesting an approximately two-fold excess of chemically non-bound oleyl phosphate ligands. The overall organic content was determined to be 32.2 wt%. Assuming that all oleic acid ligands were removed after the ligand exchange, the measured C/P-ratio of 23.2/1.0 suggests that the organic phase consists of 71 % mono- and 29 % diester. Additionally, the C/H-ratio again matches the ratio of the oleic residue of the ligand.

1. **Calculation of Oleyl Phosphate layer thickness around a spherical nanoparticle.**

*ρ_OPh_* is defined as the density of the oleyl phosphate (OPh), *ρ_NP_* as the density of the nanoparticle core, *r* the radius of the nanoparticle core, and *l* the thickness of the OPh layer around the NP core.

Therefore, assuming a perfect sphere, the mass of a NP core can be expressed as:

| $m_{NP}=\rho_{NP}\frac{4}{3}\pi r^{3}$ | (7) |
| --- | --- |

The mass of OPh in a layer of thickness *l* around the NP core is therefore calculated as:

| $m_{OPh}=\rho_{OPh}[\frac{4}{3}\pi\left( r+l \right)^{3}-\frac{4}{3}\pi r^{3})]$ | (8) |
| --- | --- |

The weight fraction of OPh (*w_OPh_)* corresponds to the relative mass of OPh in the sample, and can be expressed as:

$w_{OPh}=\frac{m_{OPh}}{(m_{OPh}+ m_{NP})}$ (9)

Hence, combining equations 7-9, the weight fraction of OPh is calculated as:

$w_{OPh}=\frac{\rho_{OPh}[\left( r+l \right)^{3}-r^{3}]}{(\rho_{OPh}[\left( r+l \right)^{3}-r^{3}]+ \rho_{NP}r^{3})}$ (10)

Equation 10 is a third order polynomial equation with three roots, being the only real root that solves for *l*:

| $l=\frac{{[r^{3}\rho_{OPh}^{2}\left( 1-w_{OPh} \right)^{2}\left[ \rho_{OPh}\left( 1-w_{OPh} \right)+\rho_{NP}w_{OPh} \right]]}^{\frac{1}{3}}}{\rho_{OPh}(1- w_{OPh})}-r$ | (11) |
| --- | --- |

Solving equation 11 with *ρ_OPh_*= 0.95 g/cm^3^, *ρ_NP_*=*ρ_Fe3O4_* = 5.24 g/cm^3^, *r* = 9 nm, and *w_OPh_* = 0,12, the thickness of the OPh layer around a 9 nm in radius NP core is 1.9 nm.

1. **SAXS of the self-assembled materials.**

**
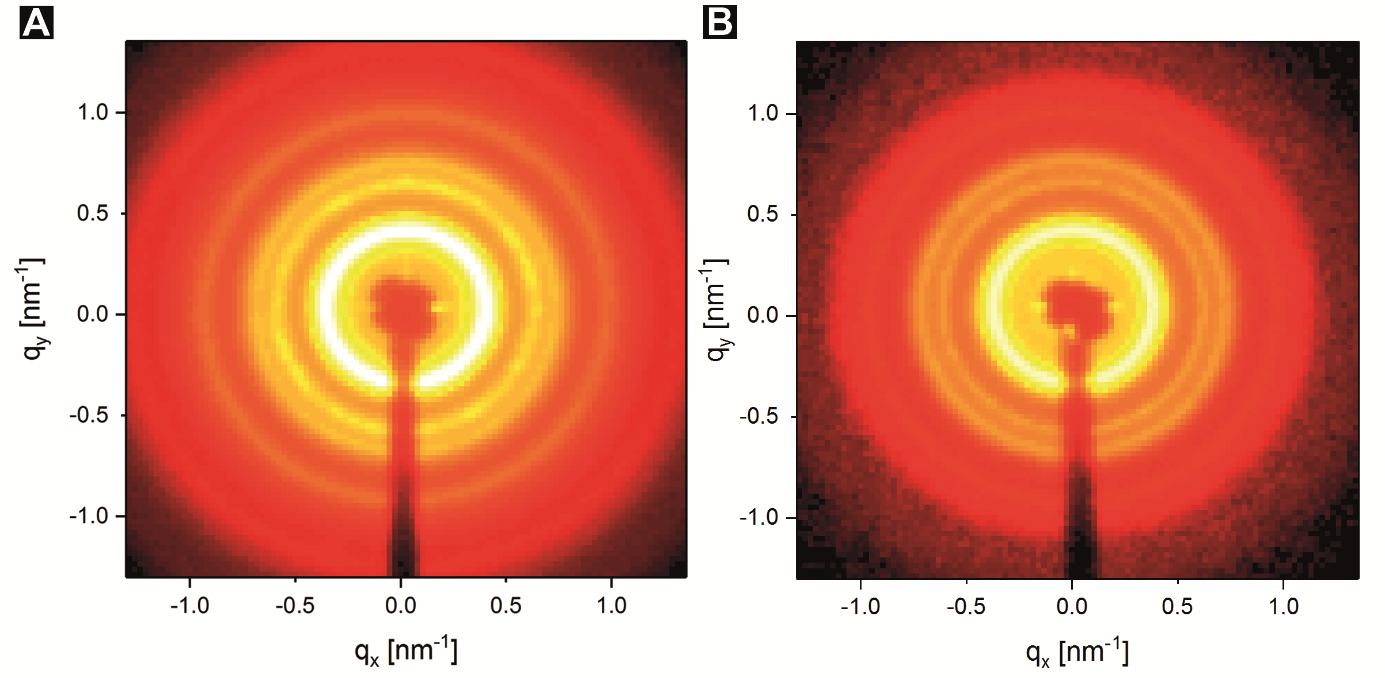
Figure SI 6.** Diffraction patterns of two samples prepared with (A) Batch 1 and (B) Batch 2.

|  |
| --- |

1. **% Volume organic phase calculation.**

It is assumed that the material consists of two phases; a supercrystalline (SC) with 12 wt% oleyl phosphate (OPh) and pure OPh-phase. In the following the determination of the volume content of both phases is presented based on the knowledge of the elemental analysis of the weight fraction *w*_OPh_ of OPh on the nanoparticles in the starting suspensions.

The volume of a FCC-unit cell with the corner length *a* is:

|  | $V_{fcc}=a^{3}$ | (12) |
| --- | --- | --- |

A unit cell consists of 4 nanoparticles with OPh shells. Hence the overall OPh-mass of *m*_OPh_ in one FCC unit cell is:

|  | $m_{OPh}=\frac{w_{OPh} m_{FO}}{1-w_{OPh}}$ | (13) |
| --- | --- | --- |

The mass of the iron oxide *m*_FO_ in the unit cell can be calculated from the density 𝜌_FO_ and the volume of the nanoparticle with a radius *r*_FO_ as:

$m_{FO}={4 \rho}_{FO}\frac{4}{3}\pi{r_{FO}}^{3}$ (14)

As it is assumed that *w*_OPhSC_=0.12 is constant in the SC and the amount of phase separated OPh *∆m*_OPh_ is

$\Delta m_{OPh}=m_{OPh}\left( w_{OPhi} \right)-m_{OPh}\left( w_{OPhSC} \right)$ (15)

where *w*_OPhi_ is the initial weight fraction of OPh. The volume *∆V*_OPh_ of this OPh phase is:

$\Delta V_{OPh}=\frac{\Delta m_{OPh}}{\varrho_{OPh}}$ (16)

Using equations 12-16, finally the volume content of the SC-phase *f*_SC_ and the OPh phase *∆f*_OPh_ is:

$\Delta f_{OPh}=\frac{\Delta V_{OPh}}{\Delta V_{OPh}+V_{SC}}=\frac{\varrho_{OPh}\rho_{FO}\frac{16}{3}\pi{r_{FO}}^{3}\left( \frac{w_{OPhi}}{{1-w}_{OPhi}}-\frac{w_{OPhSC}}{{1-w}_{OPhSC}} \right)}{\rho_{FO}\frac{16}{3}\pi{r_{FO}}^{3}\left( \frac{w_{OPhi}}{{1-w}_{OPhi}}-\frac{w_{OPhSC}}{{1-w}_{OPhSC}} \right)+{\varrho_{OPh} a}^{3}}$ (17)

$f_{SC}=1-\Delta f_{OPh}$ (18)

From SAXS measurements the unit cell length a of the FCC supercrystal is 27.5 nm for Batch 1 and 28.2 nm for Batch 2.

Hence for this investigation for Batch 2 it is: *w*_OPhi_*=0.32*, *w*_OPhSC_*=0.12,* *a=28.2nm, r*_FO_*=9.3nm*, 𝜌_FO_=5.24g/cm^3^, 𝜌_OPh_=0.95g/cm^3^

Finally, the volume percentage of the two phases turns out to be exactly 50%, *∆f*_OPh_=0.5 and *f*_SC_=0.5.

1. **Synchrotron microtomography (SRµCT).**
   1. **Estimation of the Representative Volume of Interest (RVI-test):**


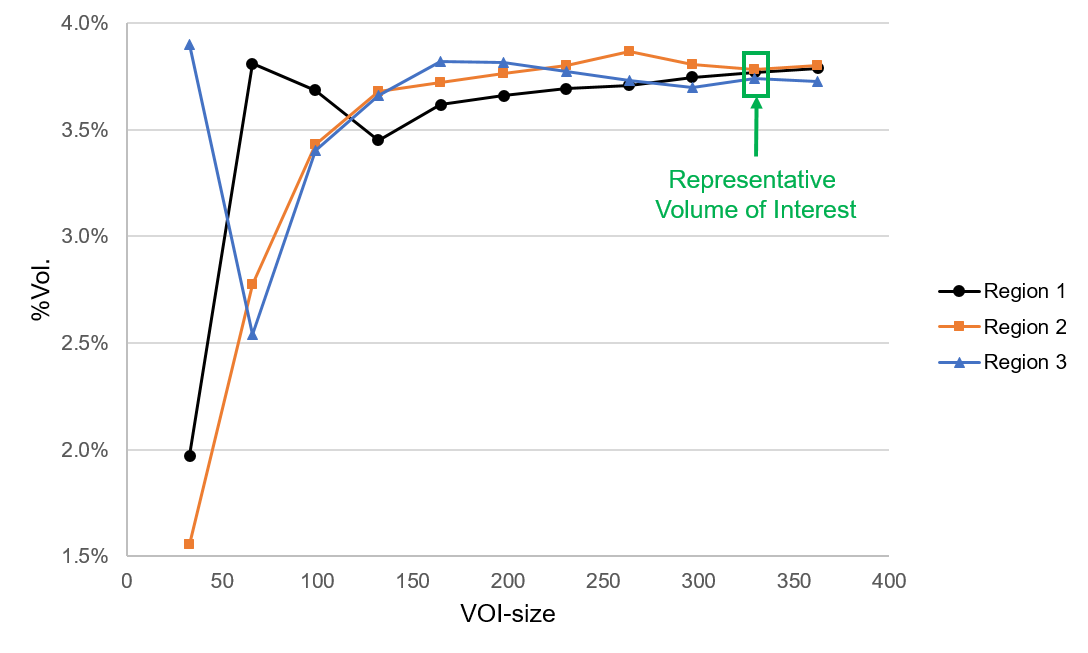


**Figure SI 7.** Test for finding the Representative Volume of Interest for VOIs selected in the outer edge of the sample. The %vol. is dependent on the chosen VOI-size. Optimal VOI-size was chosen at (332x332x332) µm^3^. The same test was used for all the other regions of the sample.

Following this test, the total amount of VOIs selected from each region was as follows: 3 VOIs from the bottom layer (Figure 5B) at a distance of 0.1 mm from the bottom of the sample; 14 VOIs from the outer edge (Figure 5C) - 4, 4, 6 VOIs along the curve of the cut sample-disk at a distance of 0.4 mm, 0.7 mm, and 1.1 mm from the bottom of the sample-; 8 VOIs from the inner part of the sample (Figure 5D) distributed over the entire region at a distance of 0.5 mm from the bottom of the sample; and 3 VOIs from the upper part of the sample (Figure 5E) at a distance of 0.1 mm, 1.0 mm, and 1.8 mm from the outer edge and at 1 mm from the bottom layer.

- 1. **Selecting the grey-level threshold**

**
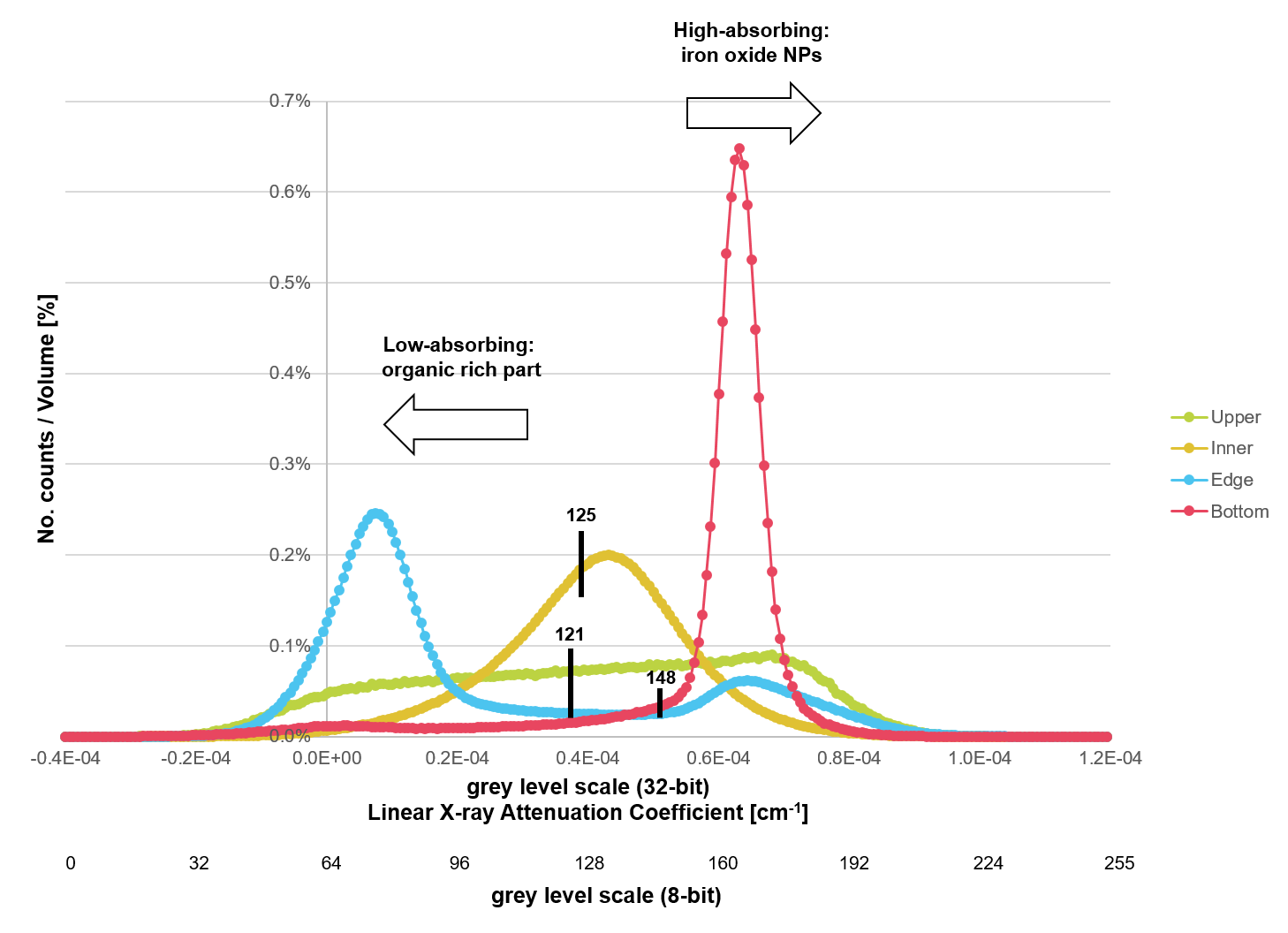
**

**Figure SI 8.** Histogram showing the grey-level distribution of a VOI from different sample specific parts of the sample: Upper, Inner, Edge and Bottom. The Y-axis has been normalized according to the equation (19). The x-axis shows both 8-bit and 32-bit grey-level scale for comparison. Thresholds were independently defined for each sample specific part following standard grey-level thresholding. The results of the thresholding procedure are highlighted in Figure SI 9.


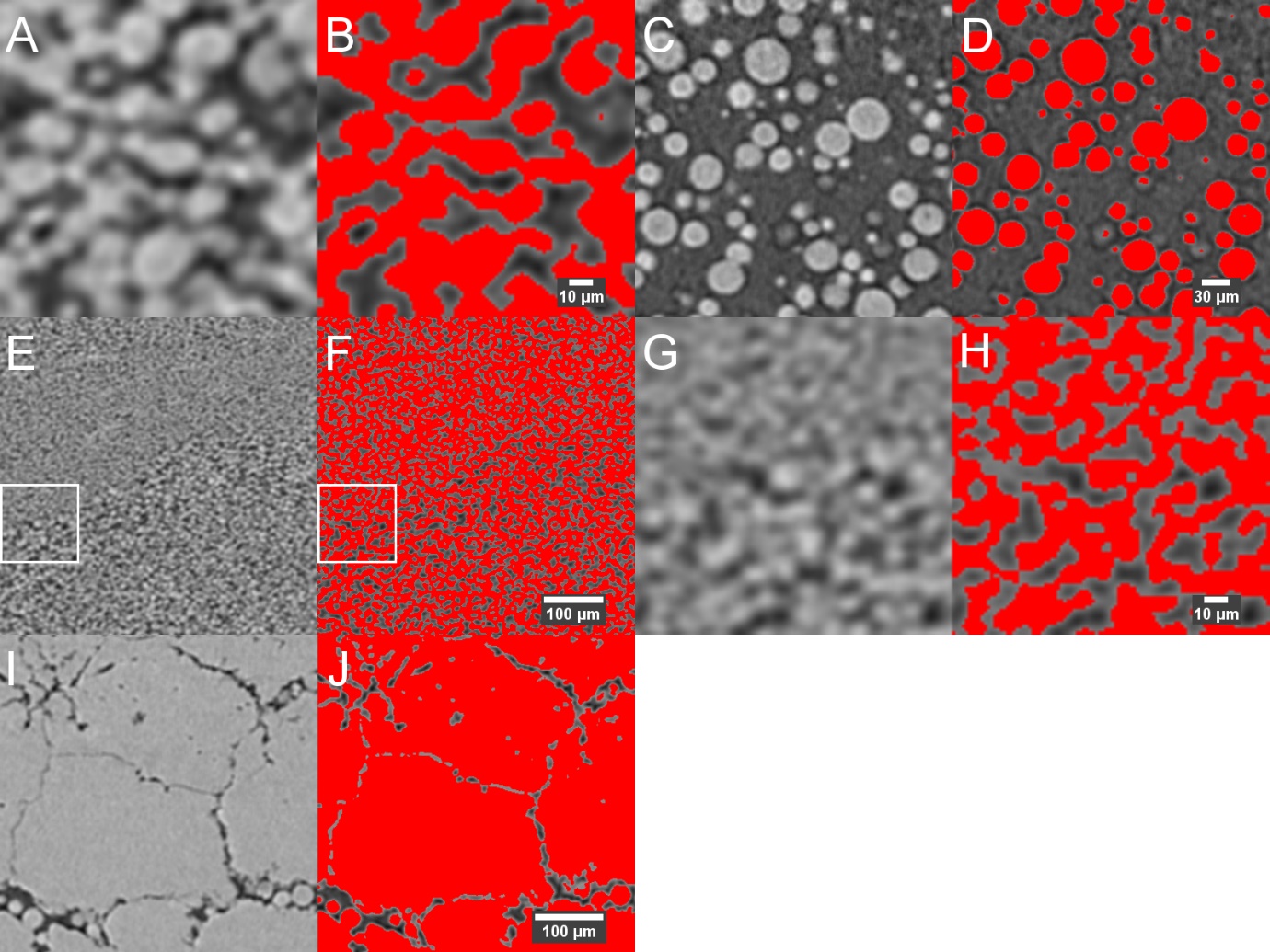


**Figure SI 9.** Regions of interests and highlighted thresholding procedure for each one of the specific parts of the sample for selecting high-absorbing the highly absorbing phase (supercrystalline) in A-B) Upper part of the sample (threshold: 121-255), C-D) Edge of the sample (threshold: 121-255), E-F) Inner part of the sample (threshold: 125-255) with highlighted rectangle marked in white G-H) zoomed-in view of E-F and I-J) Bottom of the sample (threshold: 148-255).

- 1. **Calculation of the Percentage Volume (%vol.):**

The Percentage volume (%vol.) of the high-absorbing part (supercrystalline phase) is calculated as the:

|  | $\%vol.(high abs. part)=\frac{No. of voxels of high abs. phase}{No. of total voxels of the volume}$ | (19) |
| --- | --- | --- |

where a voxel is a 3D-representation of a pixel (in 2D).

For a 2-phase material, the %vol. of the second phase, i.e. the low-absorbing phase (organic rich phase) is therefore calculated as:

|  | $\%vol \left( low abs. phase \right)=100-\%vol. (high abs. phase)$ | (20) |
| --- | --- | --- |
|  |  |  |

- 1. **Calculation of Connectivity density:**

The Connectivity density (*β*) is computed as:

|  | $\beta=\frac{\left( 1-\chi_{V} \right)}{V}$ | (21) |
| --- | --- | --- |

where $\chi_{V}$ is the Euler number defined as $\chi_{V}=(no. nodes-no. branches)$ and *V* is the volume (in mm*-3*) of the considered VOI containing the image skeleton.

- 1. **Calculation of Mean grain size and Sphericity:**

Virtual spheres (or so-called blobs) are inscribed at the nodes of the image skeleton, until each directional diameter of the sphere touches the inner border of a grain, which terminates the inscription process. Depending on the 3D structure of the grain, the inscribed blob can either have a spherical or an ellipsoidal shape. The total volume *V* (number of voxels) of each blob is calculated, followed by the estimation the radius (*r*) of the blob, according to:

|  | $r=\left( 3V/4\pi\right)^{1/3}$ | (22) |
| --- | --- | --- |

The mean grain size is then estimated as the diameter *D* of each blob, according to $D=2r$.

The sphericity of the inscribed blob is calculated as the quota between the total volume of the inscribed blob with the total volume of a so called ‘perfect sphere’ with the estimated mean diameter, *D*. If the ratio is close to the value of 1, it means that the inscribed blob is highly spherical, which is also an approximation for the quantified grain.

The exactness of the of the above proposed methods for quantifying both the mean grain size and mean grain sphericity were also verified in a second step by first applying a ‘Distance Transform Watershed 3D’ algorithm (using the MorphLibJ package^[5]^ implemented in ImageJ-Fiji^[6]^, parameters: Borgefors (3,4,5), normalization dynamic=1.) to separate connected grains from each other. In the second step, previous inscribed blobs were used as so called ‘seeding points’ to perform ‘region growing’ (IJ Plugins, Seeded Region Growing^[7]^ implemented in ImageJ-Fiji^[6]^) only of the grains laying on the longest connected skeleton axis. Hereinafter the mean grain size and mean grain sphericity were computed via ‘Blob analysis’. It was shown that the estimation of the mean grain size using the two different methods only deviated by roughly 1%, meanwhile the sphericity deviated up to roughly 10%.

The estimation of the sphericity was only performed on the VOIs from the outer edge of the sample, since both well-structured and connected grains were found here (see Figure SI9, C and D). Further investigations of sphericity were not performed for the remaining VOIs from other sample regions, i.e. upper, inner and bottom, since the majority of the grains from these regions were too well-connected and clustered (see Figure SI9 A and B, E-J and Figure SI 12), which would not provide a fair estimation of the sphericity.

**
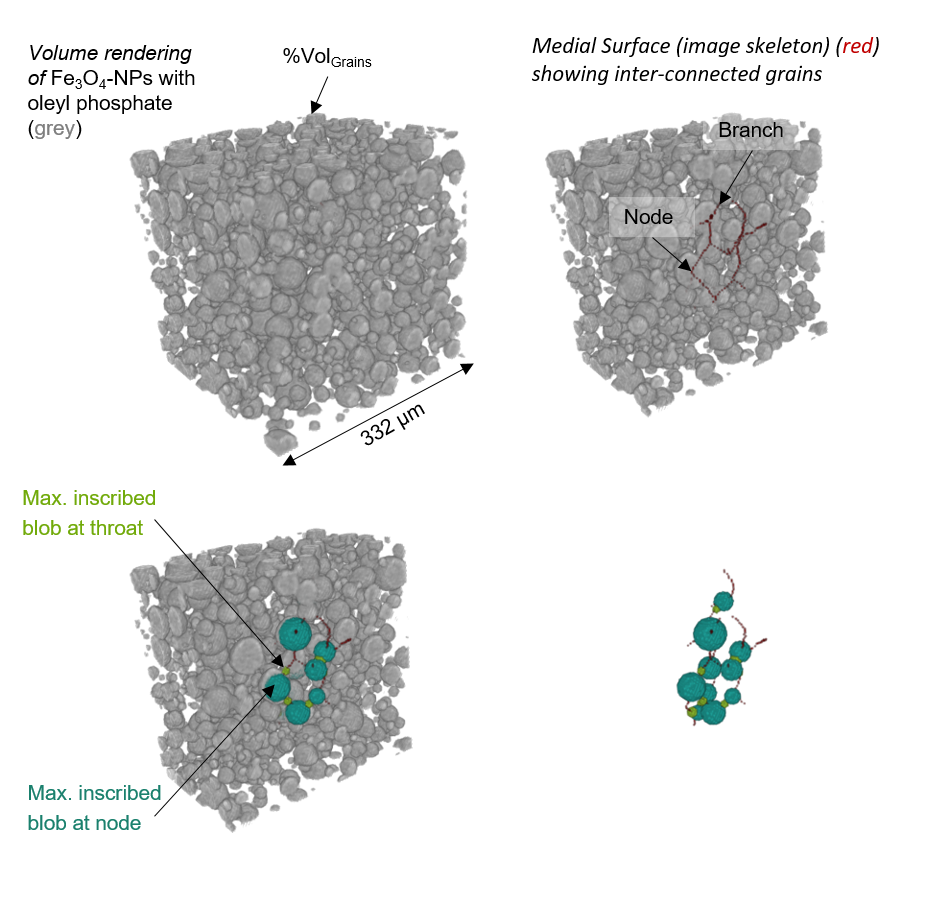
**

**Figure SI 10.** 3D-renderings a VOI (332x332x332) µm^3^ extracted from the outer edge of the sample (Figure 5C) to highlight the extracted parameters: %vol, image skeleton (branches and nodes) used for calculating the connectivity density, as well as inscribed blobs (at nodes) used for estimating the mean grain size and sphericity of the grains.

**
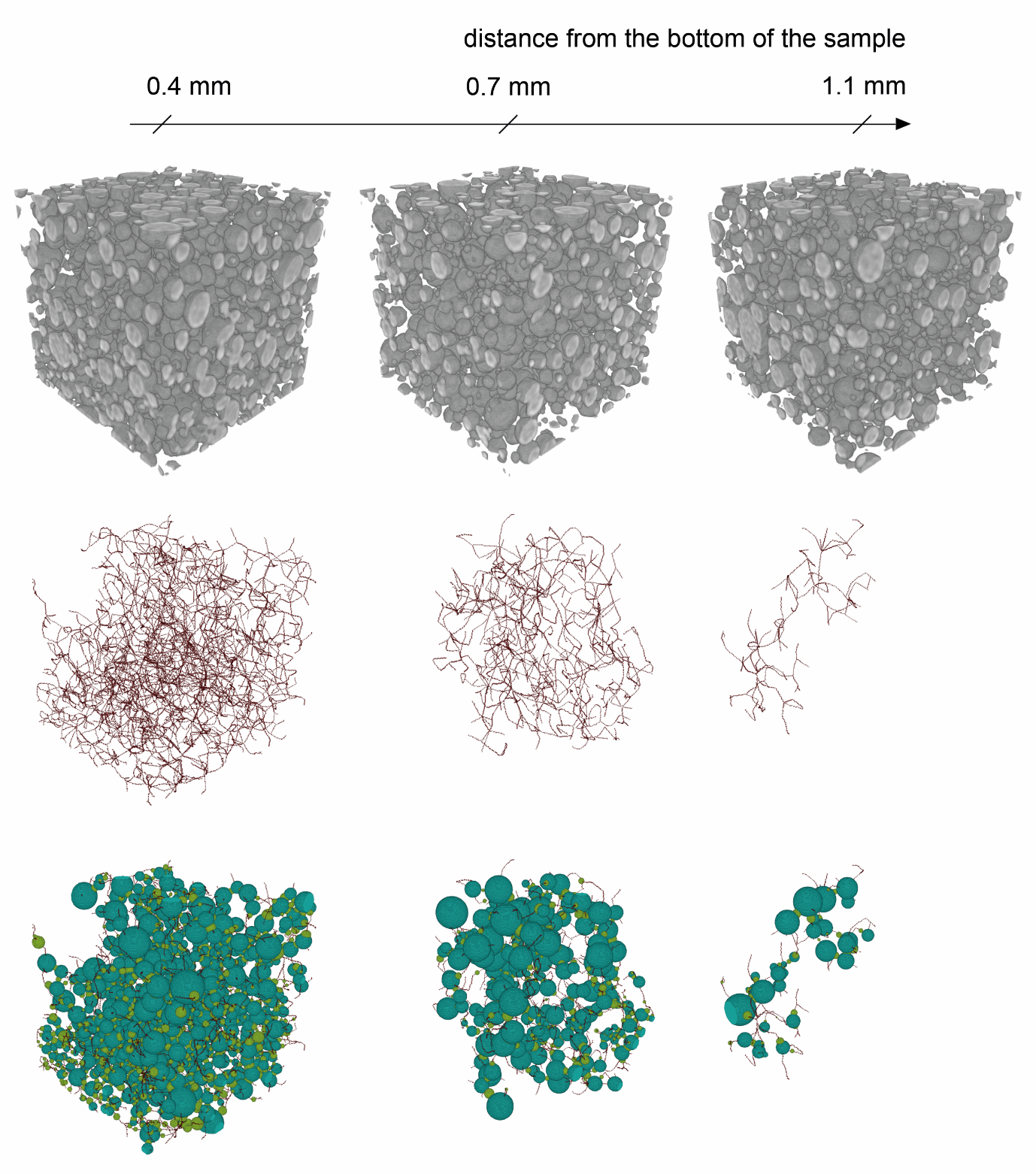
**

**Figure SI 11.** 3D-renderings of the VOIs of (332x332x332) µm^3^ extracted from the outer edge of the sample (Figure 5C) at an increasing distance from the bottom of the sample (from left to right). Upper row shows the VOIs containing grains in the range of *ca.* 23 µm. Middle row shows the longest connected 3D skeleton. Lower row shows the maximal inscribed blob inside the grains (dark green) and the maximal inscribed blob inside the necks connecting the grains together (light green).

**
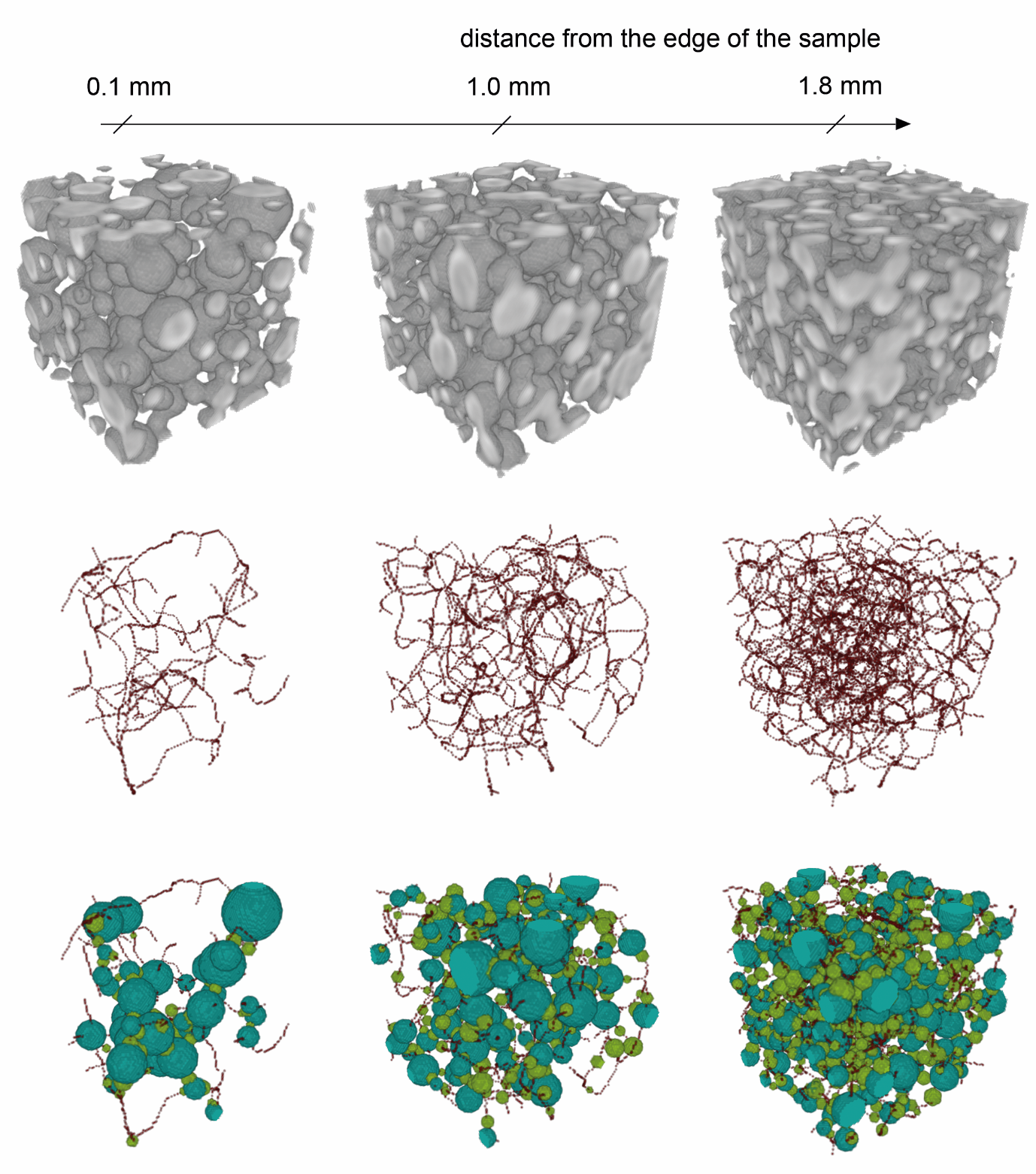
Figure SI 12.** 3D-renderings of the VOIs of (132x132x132) µm^3^ extracted from the edge of the sample (Figure 5E) at an increasing distance from the edge of the sample (from left to right). Upper row shows the VOIs containing grains in the range of *ca.* 15 µm. Middle row shows the 3D skeleton of the longest connected grains. Lower row shows the maximal inscribed blob inside the grains (dark grey) and the maximal inscribed blob inside the necks connecting the grains together (light green).

1. **References**
2. H. Borchert, E.V. Shevchenko, A. Robert, I. Mekis, A. Kornowski, G. Grübel, H. Weller, *Langmuir* **2005**, 21, 1931.
3. Y. Sahoo, H. Pizem, T. Fried, D. Golodnitsky, L. Burstein, C.N. Sukenik, G. Markovich, *Langmuir* **2001**, 17, 7907.
4. M. Rudolph, J. Erler, U.A. Peuker, U. A *Colloids Surf. A,* **2012**, 397, 16.
5. P. Roonasi, A. Holmgren, *Appl. Surf. Sci.,* **2009**, 255, 5891.
6. D. Legland, I. Arganda-Carreras, P. Andrey, *Bioinformatics*, **2016**, 32, 3532.
7. R. Adams, L. Bischof. *IEEE Trans. Pattern Anal. Mach. Intell.,* **1994**, 16, 641.
8. J. Schindelin, I. Arganda-Carreras, E. Frise, V. Kaynig, M. Longair, T. Pietzsch, S. Preibisch, C. Rueden, S. Saalfeld, B. Schmid, J.-Y. Tinevez, D. J. White, V. Hartenstein, K. Eliceiri, P. Tomancak, A. Cardona, *Nat. methods*, **2012**, 9, 676.
